# Precise photopharmacological eradication of metastatic tumor cells

**DOI:** 10.1242/dmm.052016

**Published:** 2025-02-27

**Authors:** Adam Varady, Sarah Grissenberger, Andrea Wenninger-Weinzierl, Hugo Poplimont, Caterina Sturtzel, Nicole Schmitner, Li Gao, Robin A. Kimmel, Martin Distel

**Affiliations:** ^1^ St. Anna Children's Cancer Research Institute, 1090 Vienna, Austria; ^2^ Institute of Molecular Biology/CMBI, University of Innsbruck, 6020 Innsbruck, Austria; ^3^ Ludwig Maximilian University of Munich, 80539 Munich, Germany

**Keywords:** Zebrafish xenografts, Sarcoma, Photopharmacology, Microtubules, ROS, Photodynamic therapy

## Abstract

Owing to their high efficacy, antimitotic chemotherapeutics are the mainstay for most cancer treatments. However, these drugs do not discriminate between tumor and healthy cells, thus show dose-limiting toxicity and severe adverse effects. To improve treatments, rendering chemotherapeutics tumor-cell specific is highly desirable. Although various strategies, such as targeted antibody–drug conjugates, aim to achieve this goal, the identification of a tumor-specific ‘Achilles' heel’ remains a challenge. Here, we followed an alternative approach, which does not rely on tumor-specific characteristics, but rather uses spatially confined illumination of the light-activatable microtubule inhibitor SBTubA4P to target its cytotoxic activity to tumor cells. We demonstrate that localized illumination of SBTubA4P allows for precise eradication of disseminated sarcoma cells in zebrafish xenografts without inducing systemic toxicity. In addition to the already-described light-dependent inhibition of microtubule dynamics by SBTubA4P, our data indicate that this molecule creates reactive oxygen species upon UV illumination, which significantly increases its cytotoxic effects. SBTubA4P is a valuable addition to the precision oncology toolbox, and zebrafish xenografts constitute a well-suited model to investigate photoactivatable compounds *in vivo*.

## Teaser

**Summary:** We demonstrate that SBTubA4P enables spatially precise eradication of disseminated tumor cells *in vivo* and establish zebrafish xenografts as a well-suited model for preclinical evaluation of anticancer activity of photopharmaceuticals.

## INTRODUCTION

Chemotherapeutics – including alkylating agents, antimetabolites, topoisomerase inhibitors and microtubule targeting agents – are the mainstay in current cancer treatment plans because these agents have proven to be highly efficient against tumor cells (Gascoigne and Taylor, 2009; Grünewald et al., 2018). Nevertheless, as these drugs also act on healthy cells, they have dose-limiting toxicities and generate severe adverse effects, such as cardiotoxicity and neuropathy, or can even cause secondary malignancies and infertility (Albini et al., 2010; Leone et al., 2001; Poorvu et al., 2019; Seretny et al., 2014).

Thus, one challenge for improving cancer therapy is the development of new agents with high tumor cell selectivity and minimized adverse effects on healthy tissue. Many strategies have been developed towards this goal, including targeted drugs designed to exploit cancer-specific vulnerabilities, e.g. antibody–drug conjugates, which specifically bind to tumor cells and deliver toxins or trigger phagocytosis by macrophages, and chimeric antigen receptor (CAR) T cells, which bind and lyse tumor cells (Fu et al., 2022; Hong et al., 2020; Tsimberidou et al., 2020). Although successful agents have emerged from these concepts, limiting their effects to cancer cells is still an issue, and identifying epitopes to make antibodies or CAR T cells completely cancer-cell specific remains a major challenge and area of active research. Furthermore, the heterogeneity of tumor cells makes it difficult to identify a common ‘Achilles' heel’ for targeted intervention (Dagogo-Jack and Shaw, 2018).

Photopharmacology offers an alternative strategy to limit the effects of small compounds to tumors by adding a light-responsive molecular switch to highly efficient anticancer compounds in order to control their pharmacological activity in space and time with unrivaled resolution (Hull et al., 2018; Kobauri et al., 2023). Photopharmacology and the application of light-activatable compounds is an appealing concept for precise targeting of cancer cells. To render small molecule drugs light activatable, light-sensitive ‘photocage’ motifs are often added at crucial sites of the compound to block its activity until illumination [typically, ultraviolet (UV)] irreversibly removes the cage (Klan et al., 2013; Monteiro et al., 2021). Alternatively, photoswitchable compounds can be engineered to switch from inactive to active conformations when exposed to a specific wavelength without releasing any byproducts (Gao et al., 2021, 2022). Photoswitches are often reversible in the sense that the compounds can be switched back to the inactive structure by illumination with another wavelength, or through spontaneous relaxation (Kobauri et al., 2023). Because the activity of photoswitchable drugs can be controlled with spatial and temporal precision, they can be administered in their inactive state at much higher doses than constitutively active drugs. Spatially confined activation is then able to achieve high local doses of the active compound while keeping systemic levels low (Fuchter, 2020; Hull et al., 2018; Velema et al., 2014). Importantly, the concept of using light against tumor cells is already exploited by photodynamic therapy (PDT) in the clinics (Kwiatkowski et al., 2018), with PDT specifically relying on the generation of reactive oxygen species (ROS) upon illumination of photosensitizer compounds.

Adding to the portfolio, SBTubA4P, a photoswitchable compound derived from the microtubule inhibitor combretastatin A4 phosphate (CA4P), was recently developed (Gao et al., 2022). SBTubA4P can be switched from its biologically inactive *trans-*configuration (*E*) to its active *cis-*configuration (*Z*) by UV light, which binds to tubulin and blocks polymerization of microtubules, analogous to clinically applied compounds – such as colchicine and related agents – that have entered late-stage clinical trials (e.g. combretastatin, ZD6126, ombrabulin) (Tozer et al., 2008). We previously demonstrated that localized *E-*to-*Z-*photoswitching of SBTubA4P can be applied for spatially precise short-term inhibition of microtubule polymerization *in vitro* and *in vivo* in a variety of models, including zebrafish larvae (Gao et al., 2022). Here, we explore the potential of SBTubA4P for targeted elimination of metastatic tumor cells using zebrafish xenografts as a preclinical cancer model. We chose osteosarcoma and Ewing sarcoma as tumor entities for xenograft experiments owing to the high clinical need to develop novel therapeutic strategies for patients with metastases. Our study provides evidence in larval zebrafish that metastases of pediatric sarcomas can be eradicated with SBTubA4P with high spatial precision and without adverse systemic effects.

## RESULTS

### Illumination of SBTubA4P ablates cancer cells with spatial control *in vitro*

*Z*-SBTubA4P was shown to disrupt microtubule polymerization in multiple systems *in vitro* and *in vivo*, including in zebrafish larvae, with high spatiotemporal precision and without obvious toxicity in its inactive state (Gao et al., 2022). Because microtubule targeting agents are a major group of cancer chemotherapeutics, we examined whether SBTubA4P could also be applied for spatially confined induction of cell death in cancer cells with no adverse systemic effects on normal cells. This goal has proven elusive for photoswitchable reagents based on alternative chemical scaffolds, such as the popular azobenzene photoswitches (An et al., 2019; Borowiak et al., 2015), but we considered that the greater druglikeness and metabolic stability of the styrylbenzothiazole (SBT) scaffold at the core of SBTubA4P ought to improve its chances of long-term *in vivo* efficacy (Gao et al., 2021, 2022).

**Fig. 1.**
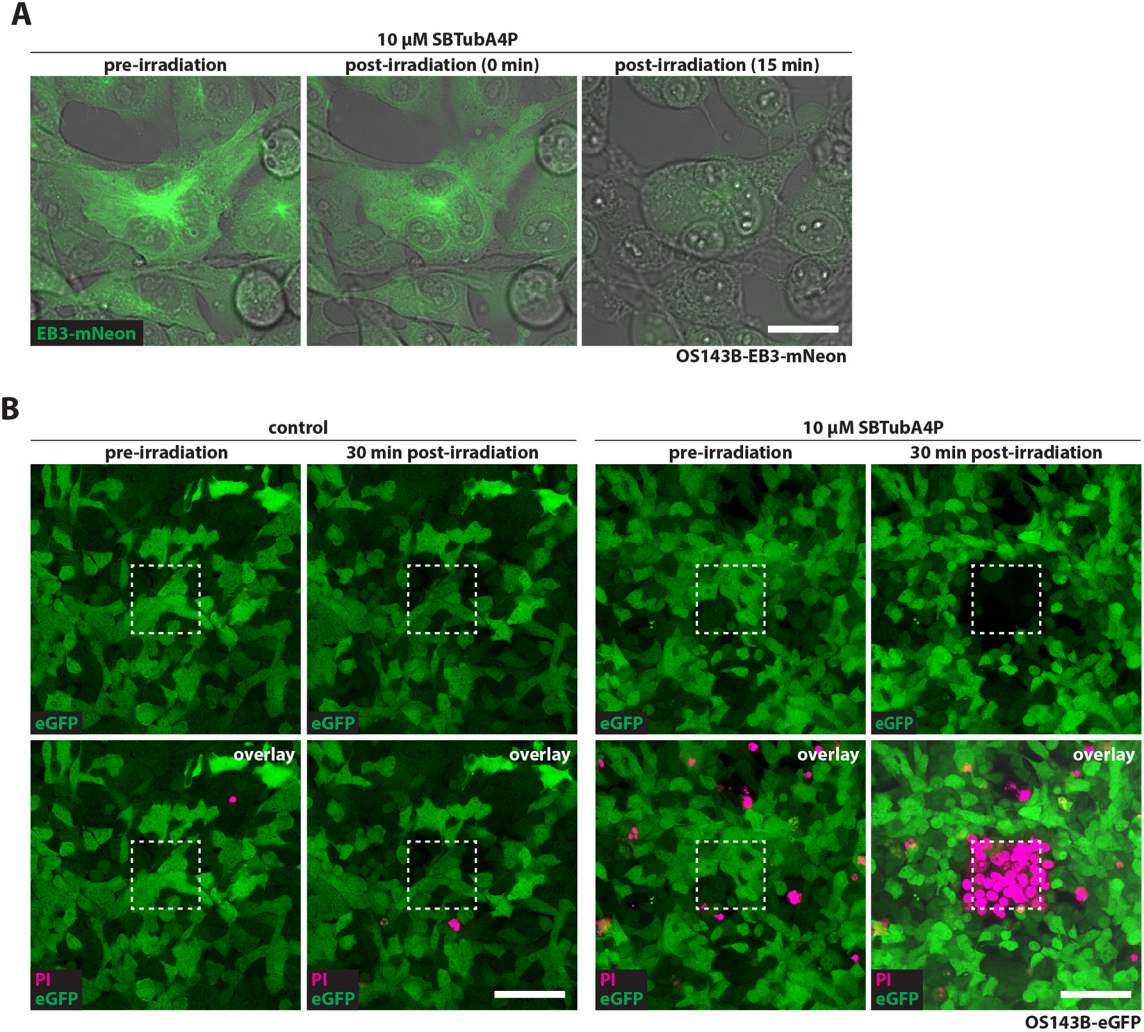
**Local illumination of SBTubA4P ablates cancer cells with spatial control *in vitro*.** (A) Repeated SBTubA4P illumination causes blebbing, contraction and swelling of cancer cells. OS143B-EB3-mNeon cells were incubated with 10 µM SBTubA4P for 1 h. Illumination was performed by repeated laser scanning with the 405 nm UV laser diode of a confocal microscope (Leica SP8 WLL) for 3 min (∼15 s between scans). Standstills from the subsequent timelapse (see Movie 2) show the onset of contraction and blebbing of cells (0 min timepoint) followed by swelling and loss of fluorescence (15 min timepoint). Representative confocal images from three independent experiments. Scale bar: 25 µm. (B) Spatially confined SBTubA4P illumination induces localized cell death in OS143B cells. OS143B-eGFP cells were incubated with 10 µM SBTubA4P for 1 h before illumination or left untreated (control). Propidium iodide (PI) solution was added to the medium before imaging. UV irradiation by repeated scanning on a confocal microscope was performed for 5 min in an area of 95×95 µm, depicted by the dashed line squares. PI staining and loss of eGFP fluorescence indicate cell death (see Movies 3 and 4). Representative confocal images (*n*=3 per condition). Scale bars: 100 µm.

First, we reproduced the transient inhibition of microtubule polymerization by photoactivated *Z*-SBTubA4P in cancer cells using the osteosarcoma cell line OS143B. To be able to observe microtubule dynamics, we transduced OS143B cells to express mNeonGreen-tagged end-binding protein EB3 (OS143B-EB3-mNeon), which fluorescently labels microtubule plus ends. After incubating OS143B-EB3-mNeon cells with 10 µM *E*-SBTubA4P, we UV irradiated specific regions using a 405 nm laser on a confocal microscope. In accordance with our previous observations in zebrafish cells, EB3-mNeon comets disappeared immediately upon UV *E*-to-*Z*-activation of SBTubA4P, but recovered within a few minutes because, being a small molecule (0.4 kDa), the photoactivated inhibitory *Z-*reagent diffuses readily out of the cell (Fig. S1A, Movie 1). As a single-pulse UV irradiation only transiently affected microtubule dynamics, we wondered whether repeated illumination of OS143B cells would lead to cell death. Indeed, with SBTubA4P, 3 min of repeated UV scanning resulted in cell blebbing, cell contraction, swelling and loss of eGFP fluorescence (Fig. 1A; Movie 2). Propidium iodide (PI) live staining revealed that permeabilization of cell membranes of OS143B cells treated with SBTubA4P occurred as early as 5 min following UV exposure. This effect was restricted to the illuminated area, with the majority of cells inside this region dying within 30-60 min (Fig. 1B; Movie 3). UV-irradiated control cells without SBTubA4P did not show any signs of cell death or PI uptake, indicating that the observed effects were SBTubA4P dependent and not UV induced (Fig. 1B; Movie 4). We confirmed these results with a second cancer cell line, Ewing Sarcoma SK-N-MC (Fig. S1B), demonstrating that spatially restricted photoelimination of cancer cells is feasible with SBTubA4P in sarcoma cells within an hour.

### SBTubA4P induces spatially confined cell death in larval zebrafish tissues

We next addressed whether SBTubA4P could be applied for spatially confined induction of cell death *in vivo*. Previously, we found that zebrafish larvae could tolerate high concentrations of inactive *E*-SBTubA4P for up to 24 h with no notable signs of toxicity (Gao et al., 2022). Following exposure to UV light, SBTubA4P-treated larvae displayed morphological abnormalities at concentrations ≥1 µM. Short-term spot irradiation of individual cells in SBTubA4P-treated zebrafish embryos triggered inhibition of microtubule dynamics followed by recovery over the course of a few minutes (Gao et al., 2022), similar to what we observed in OS143B cells (Fig. S1A).

**Fig. 2.**
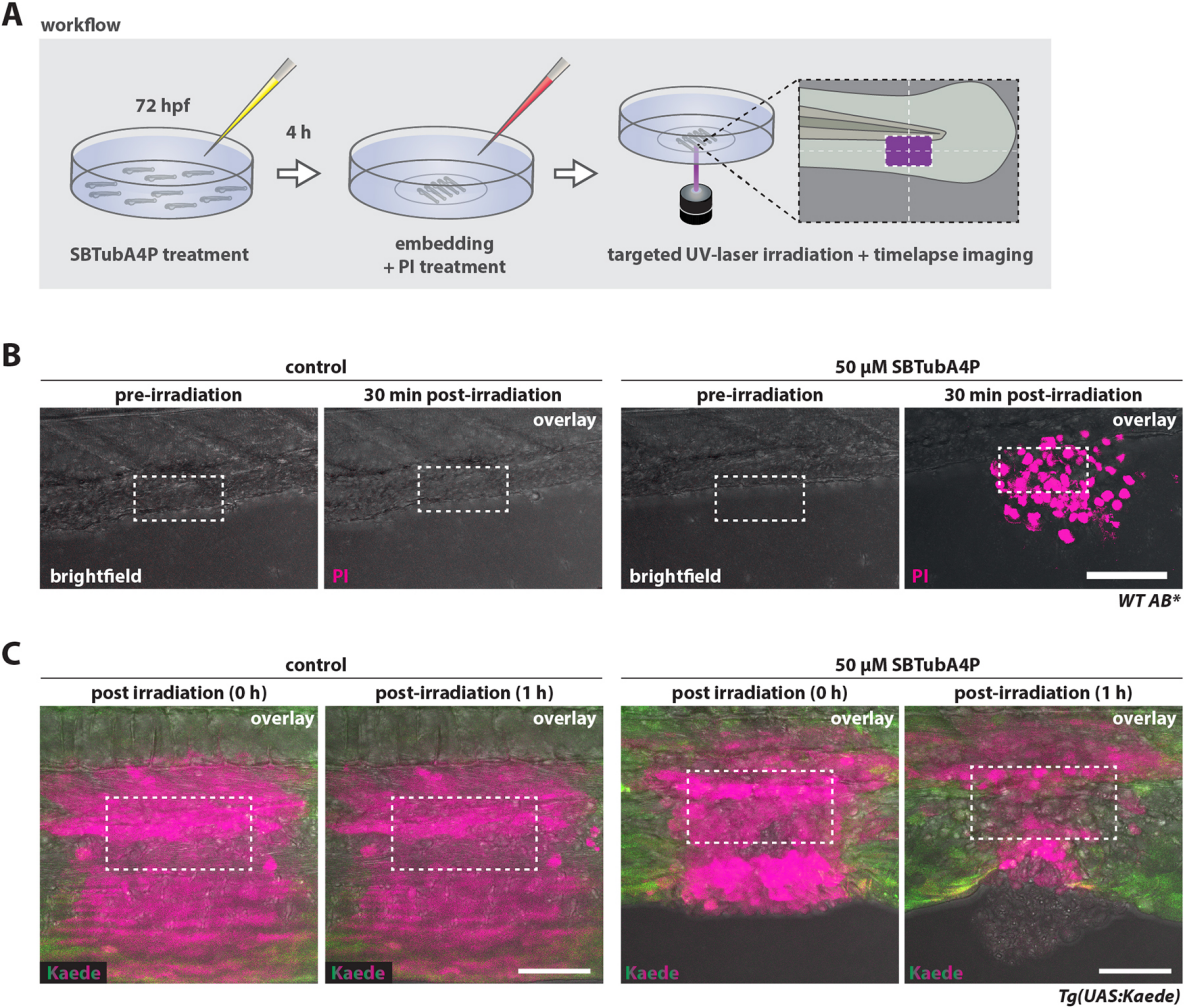
**SBTubA4P induces spatially confined cell death in larval zebrafish tissues.** (A) Schematic representation of workflow for SBTubA4P-mediated cell ablation in zebrafish larvae *in vivo*. Zebrafish larvae at 3 days post fertilization (dpf) were incubated with SBTubA4P for 4 h, anesthetized, then washed and embedded in agarose on imaging dishes. Control larvae were not treated with SBTubA4P, but otherwise underwent the same procedure. PI solution was added to the medium before imaging. Targeted illumination was performed by repeated scanning of a defined area using the UV laser diode of a Leica SP8 WLL confocal microscope. hpf, hours post fertilization. (B) Targeted cell ablation through SBTubA4P illumination in live zebrafish tissues. Wild-type (WT) AB zebrafish were treated as outlined in A, targeting an area of 95×47.5 µm (depicted by the dashed line rectangles). PI staining indicates cell death (see Movie 5). Left panel, control zebrafish without SBTubA4P; right panel, zebrafish with SBTubA4P. Representative confocal images from two independent experiments. Scale bar: 100 µm. (C) Embryos of the transgenic line *Tg(UAS:Kaede)*^rk8^ were microinjected with 25 pg KalTA4 mRNA at the one-cell stage for ubiquitous Kaede expression. At 3 dpf, larvae were treated with SBTubA4P as outlined in A but without the addition of PI. A rectangular region of interest (ROI) of 95×47.5 µm (depicted by the dashed line rectangles) was UV irradiated in control (left panel) and SBTubA4P (right panel) larvae. Red fluorescence (shown in magenta) indicates the area affected by UV through photoconversion of Kaede (see Movie 6). Representative confocal images (*n*=3 per condition). Scale bars: 50 µm.

Analogous to the rapid photoinduction of cell death in cancer cell lines *in vitro* after repeated UV illumination (Fig. 1; Fig. S1B), we went on to explore cell ablation in zebrafish larvae *in vivo*. To this end, we incubated wild-type AB zebrafish larvae at 3 days post fertilization (dpf) with 50 µM *E*-SBTubA4P in the dark for 4 h, followed by mounting for microscopy and PI addition to the fish medium. Repeated spatially confined UV illumination was performed via confocal laser scanning at 405 nm for 5 min (Fig. 2A). We observed rapid and locally confined tissue damage in SBTubA4P-treated larvae with PI uptake of dying cells within 30 min after illumination (Fig. 2B; Movie 5). Importantly, control UV-irradiated cells in the absence of SBTubA4P remained unaffected (Fig. 2B).

To determine the spatial restriction of cell death induction, we microinjected the transgenic zebrafish line *Tg(UAS:Kaede)*^rk8^ with KalTA4 mRNA for ubiquitous expression of the photoconvertible fluorescent protein Kaede, which would provide us with a readout for actual UV irradiation, including the adjacent area affected by UV light scattering (Distel et al., 2009; Hatta et al., 2006). We observed rapid onset of cell death and subsequent extrusion of cells restricted to the photoconverted Kaede area in SBTubA4P-treated larvae, whereas untreated larvae retained photoconverted red cells (shown in magenta) and did not show signs of tissue damage by UV (Fig. 2C; Movie 6).

We thereby demonstrate SBTubA4P-photomediated killing of cells to be precise and locally confined in zebrafish larvae *in vivo* without apparent cytotoxicity outside the targeted region of interest (ROI) and the adjacent margin.

### Targeted SBTubA4P illumination eliminates metastases *in vivo*

**Fig. 3.**
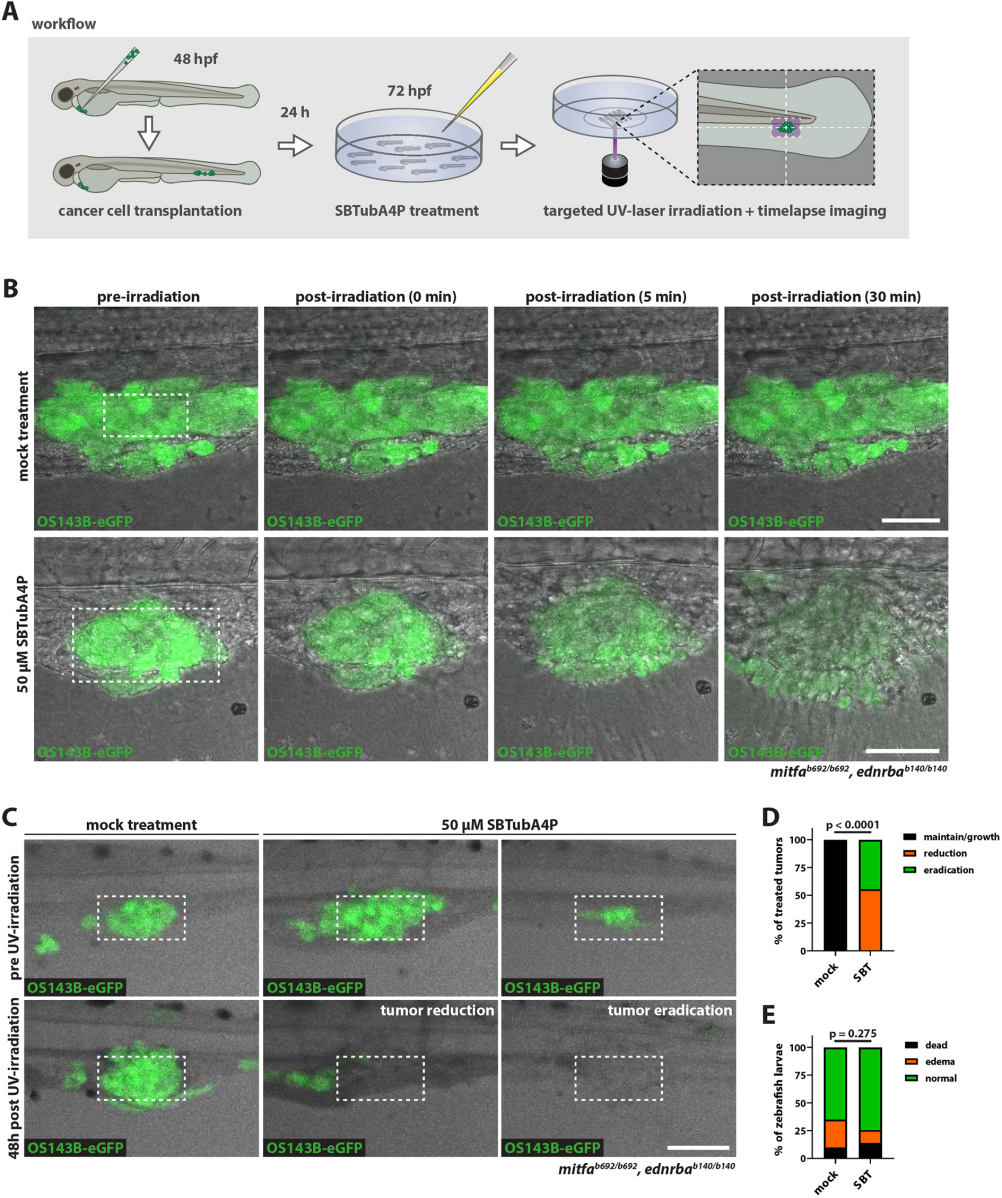
**Targeted SBTubA4P illumination eliminates metastases *in vivo*.** (A) Schematic representation of the workflow for SBTubA4P-mediated ablation of disseminated tumor cells in a zebrafish xenograft model. Transplantation of cancer cells into the perivitelline space was performed at 2 dpf. After 24 h [1 day post injection (dpi)], larvae were selected for disseminated tumor cells in the caudal hematopoietic tissue (CHT), treated with 50 µM SBTubA4P for 4 h, anesthetized, washed and mounted for spatially targeted UV irradiation. Mock-treated controls were not treated with SBTubA4P. Single microtumors in the CHT were targeted by repeated UV scanning of a rectangular ROI over the course of 10 min. (B) SBTubA4P illumination induces rapid cell death in xenografted tumor cells. OS143B-xenotransplanted zebrafish larvae at 1 dpi were treated as outlined in A. The subsequent timelapse after UV illumination reveals induction of spatially confined tissue damage at the targeted area, with loss of eGFP expression and extrusion of cells over the course of 30 min in SBTubA4P-treated larvae (see Movie 7). Dashed line rectangles indicate the targeted ROI (70×35 µm). Representative confocal images from two independent experiments (*n*=3-4 per condition). Scale bars: 50 µm. (C) SBTubA4P illumination ablates OS143B xenograft microtumors *in vivo* (see Movie 8). Zebrafish larvae at 1 dpi were treated as outlined in A, then removed from the imaging dishes, washed and kept until 5 dpf (48 h post treatment) for follow-up imaging. Dashed line rectangles indicate areas targeted by UV irradiation (140×70 µm). Representative confocal images (maximum projections: ten planes, 5 µm spacing) from three independent experiments. Scale bar: 100 µm. (D) Quantification of SBTubA4P-mediated OS143B tumor treatment efficacy. Chi-squared test was performed for comparison of mock and SBTubA4P (SBT) treatments on outcome: maintenance or growth, reduction and total eradication of tumor masses. Stacked bars represent fractions of total larvae from three independent experiments (mock, *n*=21; SBT, *n*=18). (E) Quantification of SBTubA4P treatment toxicity following xenograft tumor treatment (OS143B and SK-N-MC cells). Chi-squared test was performed for comparison of mock and SBTubA4P treatment toxicity, scoring for occurrence of dead larvae, edema formation or no adverse effects. Stacked bars represent fractions of total larvae from five independent experiments (mock, *n*=40; SBT, *n*=43).

Having confirmed photoefficacy in tumor cells and healthy cells *in vivo* in zebrafish, we next set out to investigate whether SBTubA4P is suitable for precise photoeradication of disseminated tumor cells in a zebrafish xenograft model *in vivo*. Injection of OS143B-eGFP cells into *mitfa^b692/b692^; ednrba^b140/b140^* zebrafish larvae at 2 dpf into the perivitelline space resulted in larvae with disseminated tumor cells forming metastases in the caudal hematopoietic tissue (CHT) (Fig. 3A). Injected larvae were selected for the presence of multiple individual tumor masses of similar size in the CHT. One day post injection (dpi), we incubated larvae with 50 µM SBTubA4P for 4 h before mounting on imaging dishes for targeted illumination using a confocal microscope. Small tumor masses in the CHT were specifically targeted with the 405 nm laser for 10 min. Spatially confined *in situ* illumination of SBTubA4P resulted in loss of eGFP fluorescence in targeted OS143B cells and contraction of the surrounding tissues immediately after UV irradiation (Fig. 3B; Movie 7). We observed none of these effects when disseminated cells were UV irradiated in the absence of SBTubA4P (mock treatment; Fig. 3B).

Because we found that SBTubA4P effectively photoeradicates xenografted tumor cells, we next attempted targeted ablation of single metastases in xenografts at 3 dpf by confined illumination. After UV illumination, larvae were recovered from imaging dishes, washed and kept until 5 dpf. Imaging was performed at 5 dpf to evaluate target-killing and whole-larvae toxicity of SBTubA4P compared to mock treatment (Fig. 3C-E). In SBTubA4P-treated and illuminated individuals, the size of the targeted metastasis was reduced in 55.6% (10/18), and the metastasis was completely eradicated in 44.4% (8/18), of xenografted larvae, whereas all metastases in mock-treated larvae maintained their size or proliferated further (Fig. 3C,D). We also reproduced these results in xenografts using the Ewing sarcoma cell line SK-N-MC (Fig. S2A). For this cell line, metastasis size reduction occurred in 47.4% (9/19), and complete eradication occurred in 52.6% (10/19), of SBTubA4P-treated xenografts (Fig. S2B). For the SK-N-MC control mock treatment group, we also observed regression in some cases; however, this occurred both inside and outside targeted ROIs, indicating that disseminated SK-N-MC cells are not as well maintained as OS143B cells. Treatment outcomes in both cell lines reached statistical significance comparing mock and SBTubA4P treatment (*P*<0.0001; Fig. 3D; Fig. S2B). Systemic toxicity was assessed by comparing lethality and occurrence of edema in xenografted larvae treated by SBTubA4P and illumination to that in mock-treated individuals. In the illuminated SBTubA4P-treated cohort, 14% of larvae died; 10% also died in the mock-treated cohort. Edemas occurred in 11.6% of the surviving SBTubA4P-treated individuals compared to 25% of the mock-treated individuals (Fig. 3E). Overall, we found no significant differences in toxicity between mock and SBTubA4P treatment.

We additionally performed live staining of larvae with PI during SBTubA4P illumination to visualize the timeframe of cell-death induction following UV irradiation. This revealed first uptake of PI in cells around the targeted locations within an hour, with most cells being eliminated ∼6 h after initial UV treatment (Fig. S2C, Movie 8).

In summary, *in situ* illumination of SBTubA4P enables spatially precise and highly effective non-invasive elimination of metastases *in vivo* without overt toxicity in non-targeted tissues.

### Repeated illumination of SBTubA4P leads to ROS generation and cell death

We initially anticipated that *E*-to-*Z*-photoactivated SBTubA4P would lead to cell death via mitotic arrest and apoptosis in phototargeted cells. However, the timescale required for this is much longer than the timescale of cell death we observed in our experiments with repeated UV illumination of SBTubA4P (Bates and Eastman, 2017; Borowiak et al., 2015; Lafanechere, 2022).

We went on to investigate whether treatment with CA4P, a microtubule inhibitor closely resembling *Z*-SBTubA4P, or treatment with *Z*-switched SBTubA4P would lead to similarly rapid cell death. We treated OS143B-EB3-mNeon cells with 100 nM CA4P or 10 µM *Z*-SBTubA4P. We observed that 100 nM CA4P treatment resulted in inhibition of microtubule polymerization, blebbing and round morphology of cells, but rupturing of cell membranes and subsequent swelling did not occur within the observed timeframe (1 h post treatment). Likewise, treatment of OS143B-EB3-mNeon cells with 10 µM pre-switched *Z*-SBTubA4P stopped microtubule dynamics, but did not result in cell death within 1 h, suggesting a role of illumination with UV light in induction of cell death (Fig. S3A). Indeed, additional UV irradiation of *Z*-SBTubA4P-treated, but not CA4P-treated, cells induced cell death inside the irradiated region within 30 min (Fig. S3B).

Thus, we investigated which modes of action besides microtubule inhibition might be present when UV irradiating SBTubA4P. Phenotypically, the observed blebbing, swelling, loss of membrane integrity and intact nuclear structure resembled the process of pyroptosis (Yu et al., 2021), for which ROS are major regulators (Li et al., 2023; Su et al., 2022; Wang et al., 2022; Zhang et al., 2023).

**Fig. 4.**
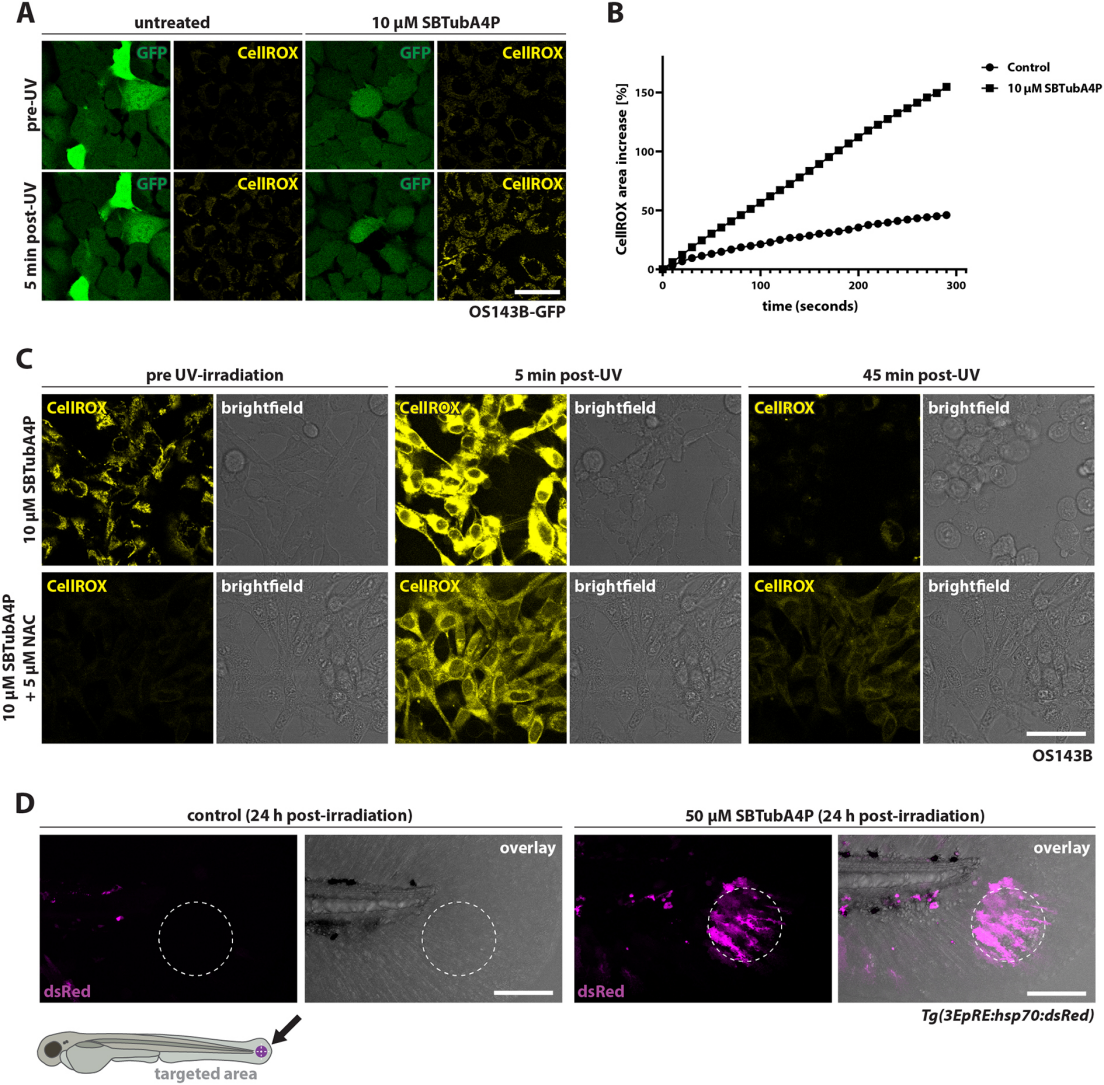
**UV illumination of SBTubA4P induces ROS.** (A,B) ROS visualization using CellROX. OS143B cells expressing GFP were treated with 10 µM SBTubA4P or left untreated 1 h before UV illumination through laser scanning for 5 min (every 10 s) in the presence of 5 µM CellROX. (A) Representative confocal images from three independent experiments. CellROX fluorescence (yellow) indicates the presence of ROS. Scale bar: 50 µm. (B) Line plot depicting the percentage increase in CellROX fluorescence area over the course of UV illumination (mean values; CellROX area divided by GFP area, normalized to the respective baseline of CellROX area). (C) Antioxidant N-acetyl-L-cysteine (NAC) reduces ROS induction by SBTuBA4P and protects cells from cell death. OS143B cells were treated with 10 µM SBTubA4P with or without 5 µM NAC for 1 h before UV illumination for 5 min in the presence of 5 µM CellROX (see Movies 9 and 10). Representative confocal images from two independent experiments. Scale bar: 25 µm. (D) Transgenic ROS reporter zebrafish strain indicates oxidative stress induced by SBTubA4P *in vivo*. 3 dpf *Tg(3EpRE:hsp70:dsRed)* larvae were treated with 50 µM SBTubA4P for 2 h or left untreated before washing, mounting on imaging dishes in agarose and targeted UV irradiation at a circular ROI of 125 µm diameter (depicted by dashed line circles). Representative confocal images (maximum projections: 15 planes, 5 µm spacing) from two experiments (*n*=10 per condition) were taken 24 h after UV irradiation. DsRed fluorescence appearing in the target region (shown in magenta) indicates response to electrophiles and oxidants. Scale bars: 100 µm.

To test for potential ROS production upon SBTubA4P illumination (e.g. photochemical singlet oxygen generation), we performed live-staining experiments in OS143B cells with the ROS-sensitive dye CellROX Deep Red Reagent. Indeed, we detected a rapid increase in intracellular ROS following UV irradiation of 10 µM SBTubA4P-treated cells. UV irradiation in the control without SBTubA4P also activated the ROS sensor, but to a lesser degree than that with SBTubA4P (Fig. 4A,B; Fig. S3C). Initially, the CellROX signal appeared to localize to mitochondria. CellROX fluorescence subsequently diffused in SBTubA4P-treated cells as they underwent cell death (Fig. S3D, Movie 9). We next examined whether an antioxidant could rescue induction of cell death. Therefore, we co-treated OS143B cells with 10 µM SBTubA4P and 5 µM N-acetyl-L-cysteine (NAC). NAC-treated OS143B cells displayed lower baseline ROS around their mitochondria and a more homogenously distributed increase in CellROX signal upon illumination, which subsided 45 min afterwards (Fig. 4C; Movie 10). Furthermore, NAC protected SBTubA4P-treated cells from blebbing, swelling and cell death altogether (Fig. 4C). Unexpectedly, we found NAC to cause uptake of PI in cells without phenotypic indications of cell death (Fig. S3E,F).

To confirm induction of the electrophilic stress response *in vivo*, which is triggered by ROS, through UV-illuminated SBTubA4P, we also made use of *Tg(3EpRE:hsp70:mCherry)* zebrafish, which visualize the activation of the Keap1–Nrf2 (also known as Nfe212a) pathway (Mourabit et al., 2019). In the evolutionarily conserved Keap1–Nrf2 pathway, Keap1 serves as a ROS sensor and inhibitor of Nrf2. In the presence of ROS, Keap1 no longer targets the transcription factor Nrf2 for proteasomal degradation. Consequently, Nrf2 accumulates in the nucleus and activates an antioxidant transcription program (Baird and Yamamoto, 2020). Upon SBTubA4P activation through UV irradiation, we observed fluorescent reporter expression in *Tg(3EpRE:hsp70:mCherry)* larvae confined to the target region in the tail. The UV-irradiated control group without SBTubA4P did not show any reporter expression (Fig. 4D).

Next, we examined whether antioxidants could also reduce tissue damage and cell death of cancer cells in xenotransplanted zebrafish. As we observed toxicity of high doses of NAC and NAC combined with SBTubA4P (Fig. S3G), we tested another widely used antioxidant that has been used in zebrafish before (Kang et al., 2019), mitoquinone mesylate (mitoQ), for cell death rescue experiments *in vivo*. We treated OS143B-GFP-xenotransplanted larvae with 50 µM SBTubA4P, with or without 500 nM mitoQ, prior to UV illumination. This resulted in rescue of SBTubA4P-mediated cell ablation in a third of illuminated larvae, whereas SBTubA4P treatment alone resulted in targeted cell ablation in all larvae (Fig. S3H).

Taken together, these findings suggest that, in our experimental setting, SBTubA4P induces cell death primarily through photogeneration of ROS and the resulting oxidative stress.

## DISCUSSION

Current chemotherapeutics including microtubule-targeting agents are efficient antitumor drugs, but they also act on healthy cells, causing severe adverse effects – including neuropathy, cardiotoxicity and secondary malignancies – especially if applied early on in life, as is the case in pediatric cancer patients. Systemic toxicity of chemotherapeutic agents also exerts dose-limiting toxicity, which, in turn, hinders effective treatment. Here, we investigated whether the photoswitchable microtubule inhibitor SBTubA4P enables spatially targeted treatment of cancer.

We found that SBTubA4P illumination can be applied to rapidly eradicate cancer cells *in vitro* with high spatial precision by repeated UV irradiation over the course of <10 min. Similarly, repeated local illumination in SBTubA4P-treated zebrafish larvae resulted in rapid cell death induction *in vivo*, which itself might be of interest for cell ablation and regeneration studies. As a preclinical *in vivo* model, we established larval zebrafish xenografts for osteosarcoma and Ewing sarcoma, which are ideally suited for optical manipulation, with a direct optical readout of effects on tumor cells and healthy host cells (Sturtzel et al., 2023). Using a metastatic xenograft model in zebrafish, we demonstrate that targeted SBTubA4P illumination for <10 min at UV intensities that are not phototoxic in the absence of the drug is sufficient to eliminate tumor cells in a spatially confined manner without causing systemic toxicity.

Interestingly, we observed cell death in less than an hour after illumination of SBTubA4P. This timeframe contrasts with our expected mechanism of death through mitotic-arrest-triggered apoptosis (Bates and Eastman, 2017; Borowiak et al., 2015; Lafanechere, 2022). Furthermore, the observed morphological changes of cells undergoing cell death did not resemble apoptosis, but pointed towards pyroptosis (Cookson and Brennan, 2001; Yu et al., 2021). The role of microtubule-destabilizing agents in the induction of pyroptosis is currently inconclusive and likely to be context dependent. Studies have suggested that colchicine inhibits pyroptosis in the context of cardiovascular disease (Li et al., 2024; Yang et al., 2020); others have reported that vincristine drives neuropathy by activating pro-pyroptotic signaling (Starobova et al., 2021). CA4P, the closest structural relative to SBTubA4P, has not been explicitly explored for induction of pyroptosis, but is known to cause blebbing, loss of cell junctions, contraction and necrosis in endothelial cells (Tozer et al., 2002). Although we observed inhibition of microtubule polymerization by illuminated SBTubA4P in sarcoma cells, it is likely not the main mechanism of cell death in our study. This is further supported by our finding that pre-switched *Z*-SBTubA4P inhibits microtubule dynamics, yet only induces cell death after additional UV irradiation (Fig. S3A,B).

In our experimental setting, we found that SBTubA4P illumination increases intracellular ROS and triggers signaling responses to oxidative damage. Abrogation of cell death through NAC *in vitro* and also mitoQ in one-third of targeted xenografts *in vivo* implies a role of oxidative stress in the mechanism of SBTubA4P-mediated killing. We therefore hypothesize that ROS production is the primary mechanism by which SBTubA4P induces cell death in the context of this work. The application of light-responsive antitumor compounds that amplify ROS production to trigger pyroptosis in cancer cells has been successfully demonstrated previously, and is seen as a strategy to simultaneously eradicate cancer cells and promote antitumor immunity (Li et al., 2023; Liu et al., 2024; Wang et al., 2022; Yu et al., 2022; Zeng et al., 2023). Furthermore, combinations of photosensitizers and microtubule-targeting agents such as vincristine have been investigated, showing potential for synergistic effects (Ma et al., 1996); however, the underlying mechanisms remain elusive. SBTubA4P is an addition to this toolbox, showing dual action as a microtubule inhibitor and photosensitizer.

A major limitation for potential clinical applications of light-activated drugs is currently the limited ability to bring light into the body. Light-delivery strategies usually consist of approaches for direct delivery using e.g. near-infrared (NIR) light, which is able to penetrate deeper into the tissue than UV. In combination with upconversion nanoparticles (UCNPs), which need to be targeted to the tumor cells, NIR can also be used to activate UV-switchable compounds, as UCNPs convert NIR back to UV (Wang and Liu, 2009). Probably, most feasible is the use of optic waveguides/optic fibers to deliver the light to the target tissue (Shabahang et al., 2018; Wang and Dong, 2020). For translation into the clinics, the development of novel photopharmacological compounds will have to go hand in hand with the development of novel light-delivery strategies (Pearson et al., 2021).

In conclusion, we demonstrate that precise eradication of metastases is possible without systemic effects in zebrafish xenografts with SBTubA4P. Our data indicate that the mode of action of SBTubA4P is mainly through photogeneration of ROS. Zebrafish xenografts are an ideal preclinical *in vivo* model to test novel light-activatable anticancer compounds, with their optical properties avoiding the light-delivery issues associated with larger-animal models, while allowing for high-quality assays, utilizing intravital imaging, fluorescent dyes and transgenic reporter strains.

## MATERIALS AND METHODS

### Cell culture

The following cell lines were used for this work: GFP-expressing SK-N-MC (SKshctrl; a kind gift from Beat Schäfer, University Children's Hospital, Zurich, Switzerland) and OS143B (kindly provided by Crystal Mackall, Stanford University, Stanford, CA, USA). SK-N-MC and OS143B cells (non-transduced, eGFP and EB3-mNeon) were cultured in RPMI 1640 medium with GlutaMAX^TM^ (Gibco, Thermo Fisher Scientific, Waltham, MA, USA), supplemented with 10% fetal bovine serum (FBS; Gibco) and 1% penicillin–streptomycin (10,000 U/ml; Gibco).

### Plasmids

The plasmid pCDH-EB3-mNeon was constructed by amplification of the EB3-mNeonGreen sequence from the EB3-mNeonGreen plasmid (Addgene ID: 98881) (Chertkova et al., 2020 preprint), followed by restriction ligation into a pCDH backbone for lentivirus production.

### Lentiviral transductions

Virus production to transduce OS143B cells with the EB3-mNeon construct was conducted using lenti-X 293T cells. Initially, lenti-X 293T cells were seeded in T25 flasks at a density of 1.5 million cells per flask in 6 ml Dulbecco's modified Eagle medium (DMEM; 31966-021, Gibco) supplemented with FBS and incubated for 18 h. The following day, a mixture of pCDH or lentiCRISPRv.2 plasmids, psPAX2 and pMD2g plasmids at a ratio of 3.3:2.7:1 was prepared in 250 µl DMEM. Prior to addition to the cells, the transfection reagent PureFection™ (System Biosciences, Palo Alto, CA, USA) was combined with the plasmid mixture at a ratio of 2:1 and incubated for 15 min at room temperature. Subsequently, the lenti-X 293T cells were washed, and their medium was replaced by DMEM, followed by addition of the transfection mixture. On the third day, the lenti-X 293T cells were washed, and their medium was changed to DMEM supplemented with 10% fetal calf serum and 1% penicillin–streptomycin. Harvesting of the lentivirus-containing medium occurred on the fourth and fifth days. The medium was centrifuged for 10 min at 500 ***g*** at 4°C, and the supernatant containing the virus was filtered through a 0.45 µm syringe filter (Nalgene™, Thermo Fisher Scientific, Waltham, MA, USA). The resulting virus-containing medium was then used to transduce OS143B cells. Finally, the transduced cells were sorted using FACSAriaFusion (BD Biosciences, Franklin Lakes, NJ, USA).

### Compounds

SBTubA4P was synthesized as described previously (Gao et al., 2022) and used at a final concentration of 10 µM for *in vitro* experiments on cultured cells or 50 µM for *in vivo* experiments on zebrafish larvae. Pre-switched SBTubA4P refers to SBTubA4P exposed to UV light for 5 min prior to treatment of cells. PI solution (Sigma-Aldrich, St Louis, MO, USA) was used at a final concentration of 5 µg/ml both *in vitro* and *in vivo*. CellROX Deep Red Reagent (Invitrogen, Waltham, MA, USA) was used at a final concentration of 5 µM *in vitro*. NAC (Sigma-Aldrich, USA) was used at a final concentration of 5 µM *in vitro* and 500 µM to 5 mM *in vivo*. mitoQ (MedChemExpress, Monmouth Junction, NJ, USA) was used at a final concentration of 500 nM *in vivo* experiments. CA4P was synthesized by the group of Oliver Thorn-Seshold (Ludwig Maximilian University, Munich, Germany; Gao et al., 2022) and used at a final concentration of 100 nM.

### Zebrafish maintenance and transgenic lines

Zebrafish (*Danio rerio*) were maintained under standard conditions (Kimmel et al., 1995; Westerfield, 2000), according to the guidelines of the local authorities under licenses GZ:565304-2014-6 and GZ:534619-2014-4. For xenotransplantation experiments, transparent zebrafish mutants (*mitfa^b692/b692^; ednrba^b140/b140^*) were used, and experiments were performed under license GZ:333989-2020-4. *ednrba^b140/b140^* is also known as *rose* and contains a mutated endothelin receptor Ba, which leads to a decrease in melanocytes and lack of iridophores in adult zebrafish (Johnson et al., 1995; Parichy et al., 2000; Rawls et al., 2001). *Tg(3EpRE:hsp70:mCherry)*, used for visualization of oxidative stress response *in vivo*, was generated by injection of the plasmid Tol2-3EpRE-hsp70-mCherry-polyA-Tol2 (Mourabit et al., 2019), generously provided by Dr Tetsuhiro Kudoh and Dr Aya Takesono (University of Exeter, Exeter, UK), along with transposase mRNA, according to standard procedures (Kawakami, 2007). Founders were identified, selected for low background and robust induction, and stable lines were established. *Tg(UAS:Kaede)* (Hatta et al., 2006) was used for photoconversion experiments.

### mRNA synthesis

Synthesis of KalTA4 mRNA was performed using a mMessage mMachine SP6 transcription kit (Thermo Fisher Scientific) after linearization of respective plasmids with NotI (New England Biolabs, Ipswich, MA, USA).

### Microinjections

For microinjection of mRNA, borosilicate glass capillaries with filament (GB100F-10, Science Products GmbH, Hofheim am Taunus, Germany) were pulled with a needle puller (P-97, Sutter Instruments, Novato, CA, USA). Needles were loaded with 3 µl DNA/mRNA injection mix (nuclease-free water, Phenol Red solution, mRNA), mounted onto a micromanipulator (M3301R, World Precision Instruments, Sarasota, FL, USA) and connected to a microinjector (FemtoJet 4i, Eppendorf, Hamburg, Germany). Droplets of ∼1 nl of the injection mix containing 25 pg mRNA were injected into zebrafish embryos at one-cell stage.

### Zebrafish xenotransplantation

*mitfa^b692/b692^; ednrba^b140/b140^* embryos were raised until 2 dpf at 28°C, dechorionated and anesthetized with 1× tricaine (0.16 g/l; Sigma-Aldrich, Hamburg, Germany), adjusted to pH 7 with 1 M Tris-HCl pH 9.5, in E3 medium. For transplantation, anesthetized larvae were aligned on a Petri dish lid coated with a solidified 2% agarose solution as described previously (Grissenberger et al., 2023; Sturtzel et al., 2023). For injection of tumor cells, borosilicate glass capillaries without filament (GB100T-8 P, Science Products GmbH), pulled with a needle puller (P-97, Sutter Instruments), were used. Needles were loaded with ∼5 μl tumor cell suspension, mounted onto a micromanipulator (M3301R, World Precision Instruments, Friedberg, Germany) and connected to a microinjector (FemtoJet 4i, Eppendorf). Cells were injected into the perivitelline space of zebrafish larvae. Xenotransplanted larvae were sorted for tumor cells at the CHT at 2 h post infection (hpi) and subsequently kept at 34°C.

### Illumination experiments and confocal imaging

One day after xenotransplantation (3 dpf), zebrafish larvae were anesthetized, incubated with SBTubA4P for 2-4 h, then washed and mounted in 1.2% low-melting agarose (Sigma-Aldrich, Germany) on #1.5 Glass Bottom Dishes (Cellvis, Mountain View, CA, USA). Cells were seeded on imaging µ-slides (ibidi, Fitchburg, WI, USA) and left to settle overnight before illumination experiments. Targeted UV irradiation on zebrafish larvae and cultured cells was performed using the 405 nm UV diode laser of a confocal microscope (SP8 WLL, Leica, Wetzlar, Germany) set to ∼4.5 µW power as measured by a Nova II power meter (Ophir, North Logan, UT, USA). A temperature control system (The Cube 2, Live Imaging Services, Basel, Switzerland) was used to perform zebrafish experiments at 28°C, cell-line experiments at 37°C and xenograft experiments at 34°C. After illumination experiments, zebrafish larvae were removed from agarose, washed and kept for follow-up imaging at indicated timepoints. All imaging was performed using the aforementioned Leica SP8 WLL confocal microscope. Generation of maximum projections, and image and video exports were done using Leica LAS X Software (Version 3.7.1). Video overlays were generated in Fiji (Schindelin et al., 2012).

### ROS quantification

CellROX fluorescence and GFP fluorescence area were measured in Fiji (Schindelin et al., 2012) for each frame of the confocal microscopy timelapses during UV illumination. Increase in CellROX area was calculated by dividing the area of CellROX by the area of GFP, normalized to the baseline (first frame) of each treatment condition.

### Statistical analysis

Statistical analysis was performed in GraphPad Prism 9. Chi-square test was employed to compare toxicity and treatment efficacy between mock treatment and SBTubA4P illumination in xenograft experiments.

## Supplementary Material

10.1242/dmm.052016_sup1 Supplementary information

## References

[DMM052016C1] Albini, A., Pennesi, G., Donatelli, F., Cammarota, R., De Flora, S. and Noonan, D. M. (2010). Cardiotoxicity of anticancer drugs: the need for cardio-oncology and cardio-oncological prevention. *J. Natl. Cancer Inst.* 102, 14-25. 10.1093/jnci/djp440 20007921 PMC2802286

[DMM052016C2] An, Y., Chen, C., Zhu, J., Dwivedi, P., Zhao, Y. and Wang, Z. (2019). Hypoxia-induced activity loss of a photo-responsive microtubule inhibitor azobenzene combretastatin A4. *Front. Chem. Sci. Eng.* 14, 880-888. 10.1007/s11705-019-1864-6

[DMM052016C3] Baird, L. and Yamamoto, M. (2020). The molecular mechanisms regulating the KEAP1-NRF2 pathway. *Mol. Cell. Biol.* 40, e00099-20. 10.1128/MCB.00099-20 32284348 PMC7296212

[DMM052016C4] Bates, D. and Eastman, A. (2017). Microtubule destabilising agents: far more than just antimitotic anticancer drugs. *Br. J. Clin. Pharmacol.* 83, 255-268. 10.1111/bcp.13126 27620987 PMC5237681

[DMM052016C5] Borowiak, M., Nahaboo, W., Reynders, M., Nekolla, K., Jalinot, P., Hasserodt, J., Rehberg, M., Delattre, M., Zahler, S., Vollmar, A. et al. (2015). Photoswitchable inhibitors of microtubule dynamics optically control mitosis and cell death. *Cell* 162, 403-411. 10.1016/j.cell.2015.06.049 26165941

[DMM052016C6] Chertkova, A. O., Mastop, M., Postma, M., van Bommel, N., van der Niet, S., Batenburg, K. L., Joosen, L., Gadella, T. W. J., Okada, Y. and Goedhart, J. (2020). Robust and bright genetically encoded fluorescent markers for highlighting structures and compartments in mammalian cells. *bioRxiv*. 10.1101/160374

[DMM052016C7] Cookson, B. T. and Brennan, M. A. (2001). Pro-inflammatory programmed cell death. *Trends Microbiol.* 9, 113-114. 10.1016/s0966-842x(00)01936-3 11303500

[DMM052016C8] Dagogo-Jack, I. and Shaw, A. T. (2018). Tumour heterogeneity and resistance to cancer therapies. *Nat. Rev. Clin. Oncol.* 15, 81-94. 10.1038/nrclinonc.2017.166 29115304

[DMM052016C9] Distel, M., Wullimann, M. F. and Koster, R. W. (2009). Optimized Gal4 genetics for permanent gene expression mapping in zebrafish. *Proc. Natl. Acad. Sci. USA* 106, 13365-13370. 10.1073/pnas.0903060106 19628697 PMC2726396

[DMM052016C10] Fu, Z., Li, S., Han, S., Shi, C. and Zhang, Y. (2022). Antibody drug conjugate: the "biological missile" for targeted cancer therapy. *Signal Transduct. Target Ther.* 7, 93. 10.1038/s41392-022-00947-7 35318309 PMC8941077

[DMM052016C11] Fuchter, M. J. (2020). On the promise of photopharmacology using photoswitches: a medicinal chemist's perspective. *J. Med. Chem.* 63, 11436-11447. 10.1021/acs.jmedchem.0c00629 32511922

[DMM052016C12] Gao, L., Meiring, J. C. M., Kraus, Y., Wranik, M., Weinert, T., Pritzl, S. D., Bingham, R., Ntouliou, E., Jansen, K. I., Olieric, N. et al. (2021). A robust, GFP-orthogonal photoswitchable inhibitor scaffold extends optical control over the microtubule cytoskeleton. *Cell Chem. Biol.* 28, 228-241.e226. 10.1016/j.chembiol.2020.11.007 33275880

[DMM052016C13] Gao, L., Meiring, J. C. M., Varady, A., Ruider, I. E., Heise, C., Wranik, M., Velasco, C. D., Taylor, J. A., Terni, B., Weinert, T. et al. (2022). *In vivo* photocontrol of microtubule dynamics and integrity, migration and mitosis, by the potent GFP-imaging-compatible photoswitchable reagents SBTubA4P and SBTub2M. *J. Am. Chem. Soc.* 144, 5614-5628. 10.1021/jacs.2c01020 35290733 PMC8972266

[DMM052016C56] Gascoigne, K. E. and Taylor, S. S. (2009). How do anti-mitotic drugs kill cancer cells? *J. Cell Sci.* 122, 2579-2585. 10.1242/jcs.039719 19625502

[DMM052016C14] Grissenberger, S., Sturtzel, C., Wenninger-Weinzierl, A., Radic-Sarikas, B., Scheuringer, E., Bierbaumer, L., Etienne, V., Nemati, F., Pascoal, S., Totzl, M. et al. (2023). High-content drug screening in zebrafish xenografts reveals high efficacy of dual MCL-1/BCL-X(L) inhibition against Ewing sarcoma. *Cancer Lett.* 554, 216028. 10.1016/j.canlet.2022.216028 36462556

[DMM052016C57] Grünewald, T. G. P., Cidre-Aranaz, F., Surdez, D., Tomazou, E. M., de Álava, E., Kovar, H., Sorensen, P. H., Delattre, O. and Dirksen, U. (2018). Ewing sarcoma. *Nat. Rev. Dis. Primers* 4, 5. 10.1038/s41572-018-0003-x 29977059

[DMM052016C15] Hatta, K., Tsujii, H. and Omura, T. (2006). Cell tracking using a photoconvertible fluorescent protein. *Nat. Protoc.* 1, 960-967. 10.1038/nprot.2006.96 17406330

[DMM052016C16] Hong, M., Clubb, J. D. and Chen, Y. Y. (2020). Engineering CAR-T cells for next-generation cancer therapy. *Cancer Cell* 38, 473-488. 10.1016/j.ccell.2020.07.005 32735779

[DMM052016C17] Hull, K., Morstein, J. and Trauner, D. (2018). In vivo photopharmacology. *Chem. Rev.* 118, 10710-10747. 10.1021/acs.chemrev.8b00037 29985590

[DMM052016C18] Johnson, S. L., Africa, D., Walker, C. and Weston, J. A. (1995). Genetic control of adult pigment stripe development in zebrafish. *Dev. Biol.* 167, 27-33. 10.1006/dbio.1995.1004 7851648

[DMM052016C19] Kang, D. M., Shin, J. I., Kim, J. B., Lee, K., Chung, J. H., Yang, H. W., Kim, K. N. and Han, Y. S. (2019). Detection of 8-oxoguanine and apurinic/apyrimidinic sites using a fluorophore-labeled probe with cell-penetrating ability. *BMC Mol. Cell Biol.* 20, 54. 10.1186/s12860-019-0236-x 31775627 PMC6881995

[DMM052016C20] Kawakami, K. (2007). Tol2: a versatile gene transfer vector in vertebrates. *Genome Biol.* 8 Suppl. 1, S7. 10.1186/gb-2007-8-s1-s7 18047699 PMC2106836

[DMM052016C21] Kimmel, C. B., Ballard, W. W., Kimmel, S. R., Ullmann, B. and Schilling, T. F. (1995). Stages of embryonic development of the zebrafish. *Dev. Dyn.* 203, 253-310. 10.1002/aja.1002030302 8589427

[DMM052016C22] Klan, P., Solomek, T., Bochet, C. G., Blanc, A., Givens, R., Rubina, M., Popik, V., Kostikov, A. and Wirz, J. (2013). Photoremovable protecting groups in chemistry and biology: reaction mechanisms and efficacy. *Chem. Rev.* 113, 119-191. 10.1021/cr300177k 23256727 PMC3557858

[DMM052016C23] Kobauri, P., Dekker, F. J., Szymanski, W. and Feringa, B. L. (2023). Rational design in photopharmacology with molecular photoswitches. *Angew. Chem. Int. Ed. Engl.* 62, e202300681. 10.1002/anie.202300681 37026576

[DMM052016C24] Kwiatkowski, S., Knap, B., Przystupski, D., Saczko, J., Kedzierska, E., Knap-Czop, K., Kotlinska, J., Michel, O., Kotowski, K. and Kulbacka, J. (2018). Photodynamic therapy - mechanisms, photosensitizers and combinations. *Biomed. Pharmacother.* 106, 1098-1107. 10.1016/j.biopha.2018.07.049 30119176

[DMM052016C25] Lafanechere, L. (2022). The microtubule cytoskeleton: an old validated target for novel therapeutic drugs. *Front. Pharmacol.* 13, 969183. 10.3389/fphar.2022.969183 36188585 PMC9521402

[DMM052016C26] Leone, G., Voso, M. T., Sica, S., Morosetti, R. and Pagano, L. (2001). Therapy related leukemias: susceptibility, prevention and treatment. *Leuk. Lymphoma* 41, 255-276. 10.3109/10428190109057981 11378539

[DMM052016C27] Li, M., Kim, J., Rha, H., Son, S., Levine, M. S., Xu, Y., Sessler, J. L. and Kim, J. S. (2023). Photon-controlled pyroptosis activation (PhotoPyro): an emerging trigger for antitumor immune response. *J. Am. Chem. Soc.* 145, 6007-6023. 10.1021/jacs.3c01231 36881923 PMC12962054

[DMM052016C28] Li, H., Yang, H., Qin, Z., Wang, Q. and Li, L. (2024). Colchicine ameliorates myocardial injury induced by coronary microembolization through suppressing pyroptosis via the AMPK/SIRT1/NLRP3 signaling pathway. *BMC Cardiovasc. Disord.* 24, 23. 10.1186/s12872-023-03697-8 38172692 PMC10765930

[DMM052016C29] Liu, J., Chen, T., Liu, X., Li, Z. and Zhang, Y. (2024). Engineering materials for pyroptosis induction in cancer treatment. *Bioact. Mater.* 33, 30-45. 10.1016/j.bioactmat.2023.10.027 38024228 PMC10654002

[DMM052016C30] Ma, L. W., Berg, K., Danielsen, H. E., Kaalhus, O., Iani, V. and Moan, J. (1996). Enhanced antitumour effect of photodynamic therapy by microtubule inhibitors. *Cancer Lett.* 109, 129-139. 10.1016/s0304-3835(96)04437-0 9020912

[DMM052016C31] Monteiro, D. C. F., Amoah, E., Rogers, C. and Pearson, A. R. (2021). Using photocaging for fast time-resolved structural biology studies. *Acta Crystallogr. D Struct. Biol.* 77, 1218-1232. 10.1107/S2059798321008809 34605426 PMC8489231

[DMM052016C32] Mourabit, S., Fitzgerald, J. A., Ellis, R. P., Takesono, A., Porteus, C. S., Trznadel, M., Metz, J., Winter, M. J., Kudoh, T. and Tyler, C. R. (2019). New insights into organ-specific oxidative stress mechanisms using a novel biosensor zebrafish. *Environ. Int.* 133, 105138. 10.1016/j.envint.2019.105138 31645010

[DMM052016C33] Parichy, D. M., Mellgren, E. M., Rawls, J. F., Lopes, S. S., Kelsh, R. N. and Johnson, S. L. (2000). Mutational analysis of endothelin receptor b1 (rose) during neural crest and pigment pattern development in the zebrafish Danio rerio. *Dev. Biol.* 227, 294-306. 10.1006/dbio.2000.9899 11071756

[DMM052016C34] Pearson, S., Feng, J. and del Campo, A. (2021). Lighting the path: light delivery strategies to activate photoresponsive biomaterials in vivo. *Adv. Funct. Mater.* 31, 50. 10.1002/adfm.202105989

[DMM052016C35] Poorvu, P. D., Frazier, A. L., Feraco, A. M., Manley, P. E., Ginsburg, E. S., Laufer, M. R., LaCasce, A. S., Diller, L. R. and Partridge, A. H. (2019). Cancer treatment-related infertility: a critical review of the evidence. *JNCI Cancer Spectr.* 3, pkz008. 10.1093/jncics/pkz008 31360893 PMC6649805

[DMM052016C36] Rawls, J. F., Mellgren, E. M. and Johnson, S. L. (2001). How the zebrafish gets its stripes. *Dev. Biol.* 240, 301-314. 10.1006/dbio.2001.0418 11784065

[DMM052016C37] Schindelin, J., Arganda-Carreras, I., Frise, E., Kaynig, V., Longair, M., Pietzsch, T., Preibisch, S., Rueden, C., Saalfeld, S., Schmid, B. et al. (2012). Fiji: an open-source platform for biological-image analysis. *Nat. Methods* 9, 676-682. 10.1038/nmeth.2019 22743772 PMC3855844

[DMM052016C38] Seretny, M., Currie, G. L., Sena, E. S., Ramnarine, S., Grant, R., MacLeod, M. R., Colvin, L. A. and Fallon, M. (2014). Incidence, prevalence, and predictors of chemotherapy-induced peripheral neuropathy: a systematic review and meta-analysis. *Pain* 155, 2461-2470. 10.1016/j.pain.2014.09.020 25261162

[DMM052016C39] Shabahang, S., Kim, S. and Yun, S. H. (2018). Light-guiding biomaterials for biomedical applications. *Adv. Funct. Mater.* 28, 1706635. 10.1002/adfm.201706635 31435205 PMC6703841

[DMM052016C40] Starobova, H., Monteleone, M., Adolphe, C., Batoon, L., Sandrock, C. J., Tay, B., Deuis, J. R., Smith, A. V., Mueller, A., Nadar, E. I. et al. (2021). Vincristine-induced peripheral neuropathy is driven by canonical NLRP3 activation and IL-1beta release. *J. Exp. Med.* 218, e20201452. 10.1084/jem.20201452 33656514 PMC7933984

[DMM052016C41] Sturtzel, C., Grissenberger, S., Bozatzi, P., Scheuringer, E., Wenninger-Weinzierl, A., Zajec, Z., Dernovsek, J., Pascoal, S., Gehl, V. and Kutsch, A. (2023). Refined high-content imaging-based phenotypic drug screening in zebrafish xenografts. *NPJ Precis Oncol.* 7, 44. 10.1038/s41698-023-00386-9 37202469 PMC10195872

[DMM052016C42] Su, X., Wang, W. J., Cao, Q., Zhang, H., Liu, B., Ling, Y., Zhou, X. and Mao, Z. W. (2022). A Carbonic anhydrase IX (CAIX)-anchored rhenium(I) photosensitizer evokes pyroptosis for enhanced anti-tumor immunity. *Angew. Chem. Int. Ed. Engl.* 61, e202115800. 10.1002/anie.202115800 34842317

[DMM052016C43] Tozer, G. M., Kanthou, C. and Chaplin, D. J. (2008). Vascular disrupting agents in cancer therapy. In *Tumor Angiogenesis* (ed. D. Marmé and N. Fusenig), pp. 809-829. Springer.

[DMM052016C44] Tozer, G. M., Kanthou, C., Parkins, C. S. and Hill, S. A. (2002). The biology of the combretastatins as tumour vascular targeting agents. *Int. J. Exp. Pathol.* 83, 21-38. 10.1046/j.1365-2613.2002.00211.x 12059907 PMC2517662

[DMM052016C45] Tsimberidou, A. M., Fountzilas, E., Nikanjam, M. and Kurzrock, R. (2020). Review of precision cancer medicine: Evolution of the treatment paradigm. *Cancer Treat. Rev.* 86, 102019. 10.1016/j.ctrv.2020.102019 32251926 PMC7272286

[DMM052016C46] Velema, W. A., Szymanski, W. and Feringa, B. L. (2014). Photopharmacology: beyond proof of principle. *J. Am. Chem. Soc.* 136, 2178-2191. 10.1021/ja413063e 24456115

[DMM052016C47] Wang, F. and Liu, X. (2009). Recent advances in the chemistry of lanthanide-doped upconversion nanocrystals. *Chem. Soc. Rev.* 38, 976-989. 10.1039/b809132n 19421576

[DMM052016C48] Wang, J. and Dong, J. (2020). Optical waveguides and integrated optical devices for medical diagnosis, health monitoring and light therapies. *Sensors* 20, 14. 10.3390/s20143981 PMC7411870 32709072

[DMM052016C49] Wang, H., Jing, G., Niu, J., Yang, L., Li, Y., Gao, Y., Wang, H., Xu, X., Qian, Y. and Wang, S. (2022). A mitochondria-anchored supramolecular photosensitizer as a pyroptosis inducer for potent photodynamic therapy and enhanced antitumor immunity. *J. Nanobiotechnol.* 20, 513. 10.1186/s12951-022-01719-9 PMC9719646 36463229

[DMM052016C50] Westerfield, M. (2000). The zebrafish book. In *A Guide for the Laboratory Use of Zebrafish (Danio rerio)*, 4th edn. University of Oregon Press.

[DMM052016C51] Yang, M., Lv, H., Liu, Q., Zhang, L., Zhang, R., Huang, X., Wang, X., Han, B., Hou, S., Liu, D. et al. (2020). Colchicine alleviates cholesterol crystal-induced endothelial cell pyroptosis through activating AMPK/SIRT1 pathway. *Oxid. Med. Cell Longev.* 2020, 9173530. 10.1155/2020/9173530 32733639 PMC7378601

[DMM052016C52] Yu, P., Zhang, X., Liu, N., Tang, L., Peng, C. and Chen, X. (2021). Pyroptosis: mechanisms and diseases. *Signal Transduct Target Ther.* 6, 128. 10.1038/s41392-021-00507-5 33776057 PMC8005494

[DMM052016C53] Yu, L., Xu, Y., Pu, Z., Kang, H., Li, M., Sessler, J. L. and Kim, J. S. (2022). Photocatalytic superoxide radical generator that induces pyroptosis in cancer cells. *J. Am. Chem. Soc.* 144, 11326-11337. 10.1021/jacs.2c03256 35708298

[DMM052016C54] Zeng, S., Chen, C., Zhang, L., Liu, X., Qian, M., Cui, H., Wang, J., Chen, Q. and Peng, X. (2023). Activation of pyroptosis by specific organelle-targeting photodynamic therapy to amplify immunogenic cell death for anti-tumor immunotherapy. *Bioact. Mater.* 25, 580-593. 10.1016/j.bioactmat.2022.07.016 37056275 PMC10087757

[DMM052016C55] Zhang, Y., Doan, B. T. and Gasser, G. (2023). Metal-based photosensitizers as inducers of regulated cell death mechanisms. *Chem. Rev.* 123, 10135-10155. 10.1021/acs.chemrev.3c00161 37534710

