# Supplementary material for: Precise photopharmacological eradication of metastatic tumor cells
Source: Dis Model Mech. 2025 Feb 27;18(2):DMM052016. doi: 10.1242/dmm.052016 (PMC11892682; doi:10.1242/dmm.052016)
Supplement: Supplementary information [file dmm-18-052016-s1.pdf]

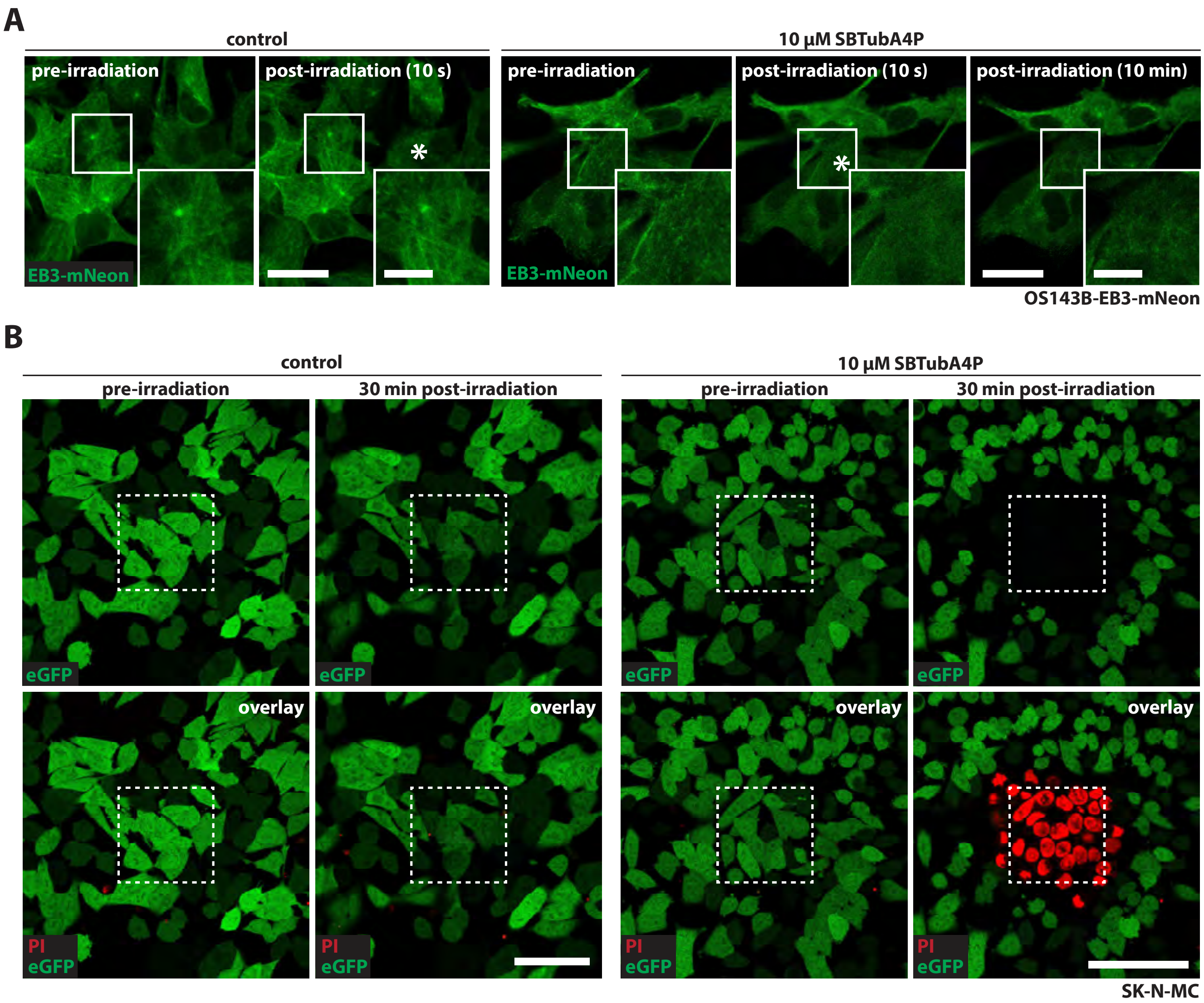

Fig. S1. Illuminated SBTubA4P inhibits microtubule polymerization and induces cell death.

(A) SBTubA4P *E*-to-*Z*-photoactivation transiently inhibits microtubule dynamics. OS143B-EB3-mNeon cells were incubated with SBTubA4P for 1 hour. Photoactivation was performed by a single 100 ms bleachpoint (indicated by the asterisk) using a 405 nm UV laser diode of a confocal microscope. No effect on EB3-comets was observed post-irradiation in untreated control cells (left panel). Loss of EB3 comets was seen immediately after photoactivation of SBTubA4P (right panel) with recovery over the course of 10 minutes (see Movie 1). Representative confocal images from 2 independent experiments. Scale bars: 25 µm or 10 µm for insets.

(B) Spatially confined SBTubA4P illumination induces localized cell death in SK-N-MC cells. SK-N-MC cells were incubated with 10 µM SBTubA4P for 1 hour before illumination (right panel) or left untreated (control - left panel). PI was added to the medium before imaging. UV-irradiation by repeated scanning on a confocal microscope was performed for 5 minutes in an area of 95 x 95 µm, depicted by the dashed square. PI staining and loss of eGFP fluorescence indicate cell death. Representative confocal images (n = 3 per condition). Scale bars: 100 µm.

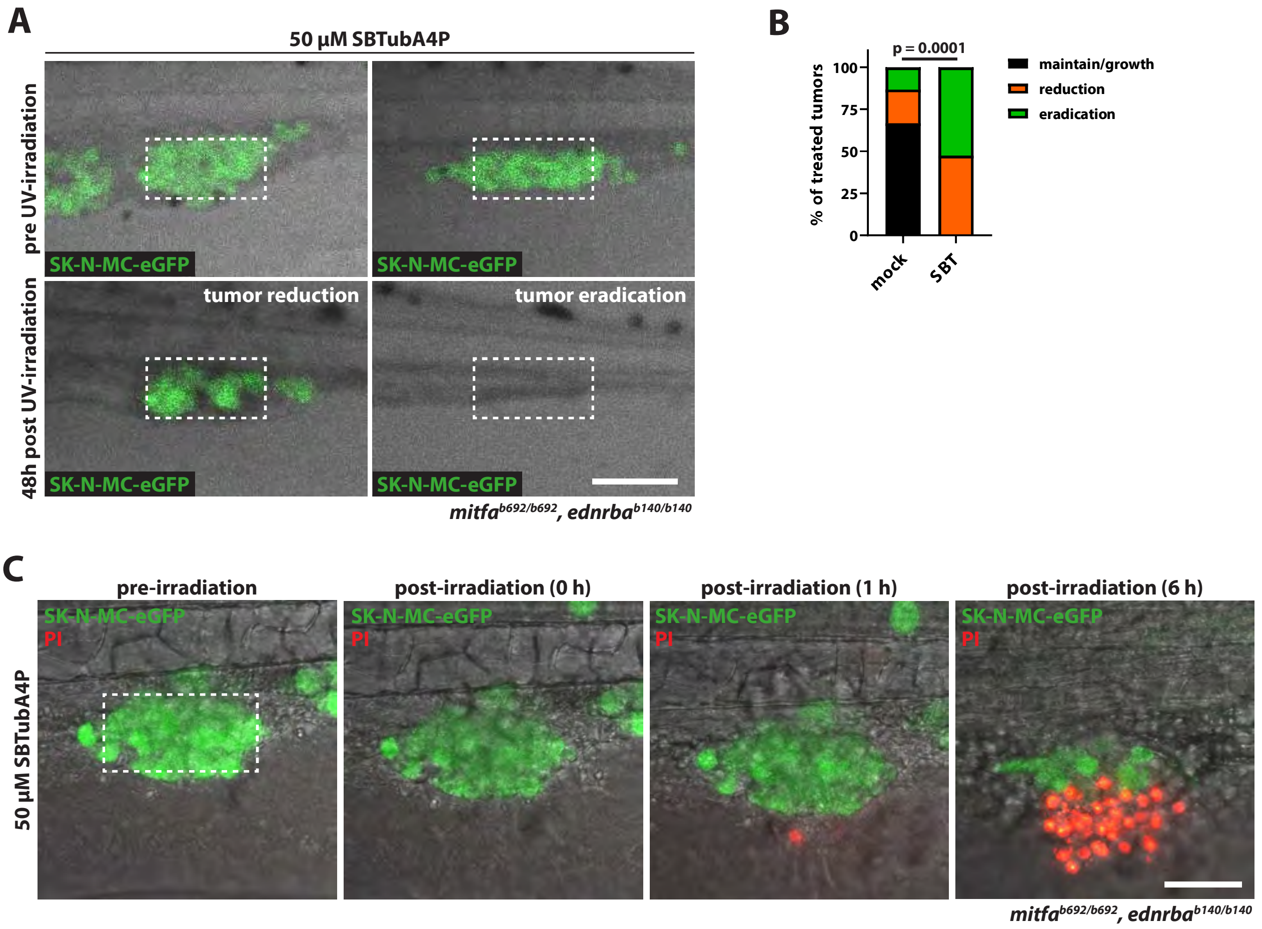

Fig. S2. Rapid induction of tissue damage and tumor ablation using SBTubA4P

(A) SBTubA4P illumination ablates disseminated SK-N-MC tumor cells *in vivo*. SK-N-MC-xenotransplanted zebrafish larvae at 1 dpi were treated with SBTubA4P as outlined in (Fig. 3A), then removed after UV illumination from the imaging dishes, washed and kept until 5 dpf (48 hours post-treatment) for follow-up imaging. Dashed rectangles depict areas targeted for UV-irradiation. Representative confocal images (maximum projections: 10 planes, 5 µm spacing) from 2 independent experiments.

(B) Quantification of SBTubA4P-mediated SK-N-MC metastases ablation efficacy. Chi-squared test performed for comparison of mock and SBTubA4P (SBT) treatments on outcome: maintenance or growth, reduction and total eradication of tumor masses. Stacked bars represent fractions of total larvae from 3 independent experiments (mock: n = 15; SBT: n = 19).

(C) PI live-staining in SBTubA4P-mediated SK-N-MC tumor treatment. SK-N-MC-xenotransplanted zebrafish larvae at 1 dpi were treated as outlined in (A) with PI solution added after embedding in imaging dishes and time-lapse imaging performed for 6 hours after UV-irradiation. Dashed rectangle in pre-irradiation panel depicts targeted ROI (95 x 47.5 µm). PI uptake indicates cell death. Representative confocal images (maximum projections: 9 planes, 5 µm spacing) for n = 5. Scale bar: 50 µm.

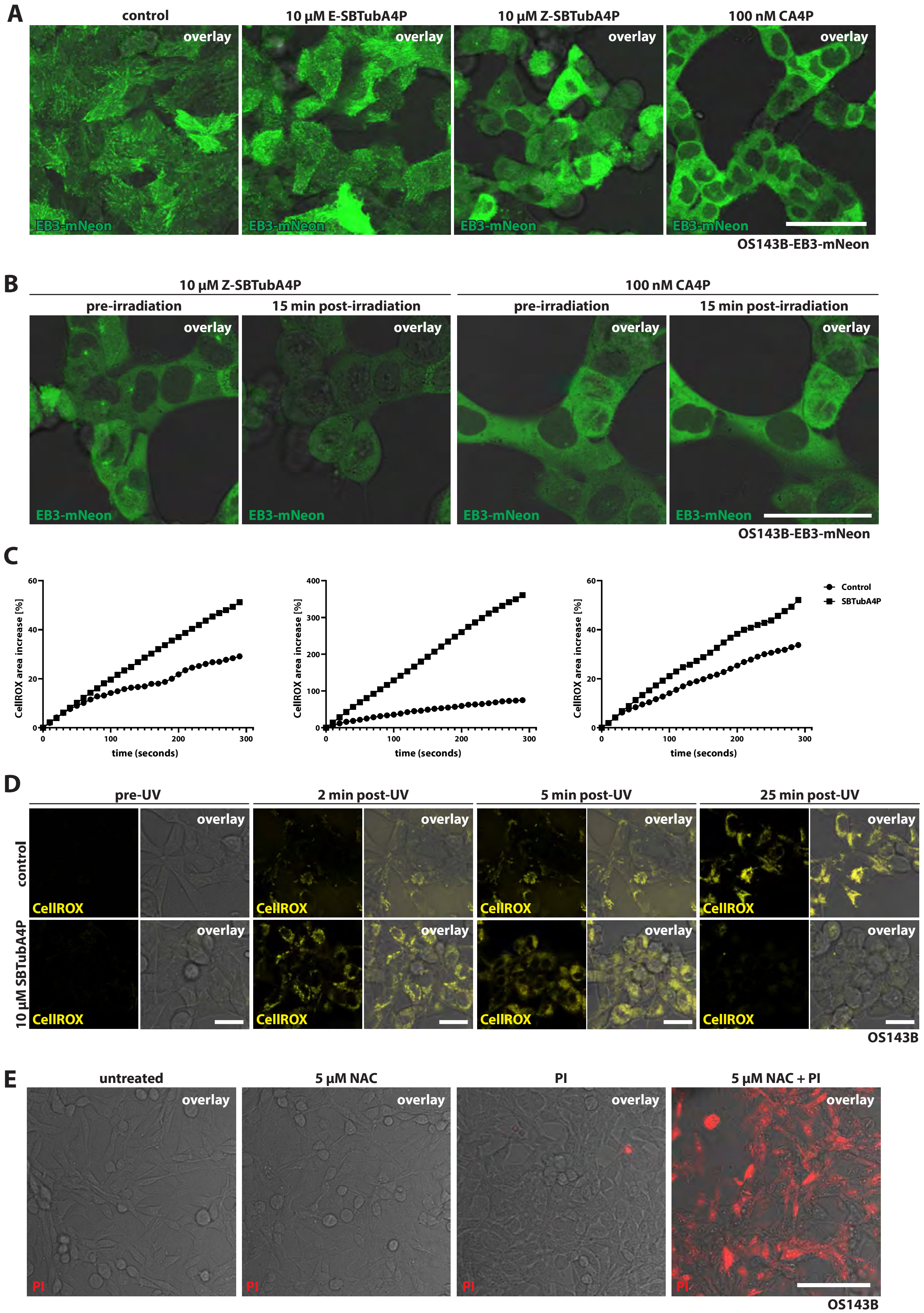
A
control
10 µM E-SBTubA4P
10 µM Z-SBTubA4P
100 nM CA4P
overlay
EB3-mNeon
OS143B-EB3-mNeon
B
10 µM Z-SBTubA4P
100 nM CA4P
pre-irradiation
15 min post-irradiation
overlay
EB3-mNeon
OS143B-EB3-mNeon
C
CellROX area increase [%]
time (seconds)
Control
SBTubA4P
D
pre-UV
2 min post-UV
5 min post-UV
25 min post-UV
control
10 µM SBTubA4P
CellROX
overlay
OS143B
E
untreated
5 µM NAC
PI
5 µM NAC + PI
overlay
OS143B

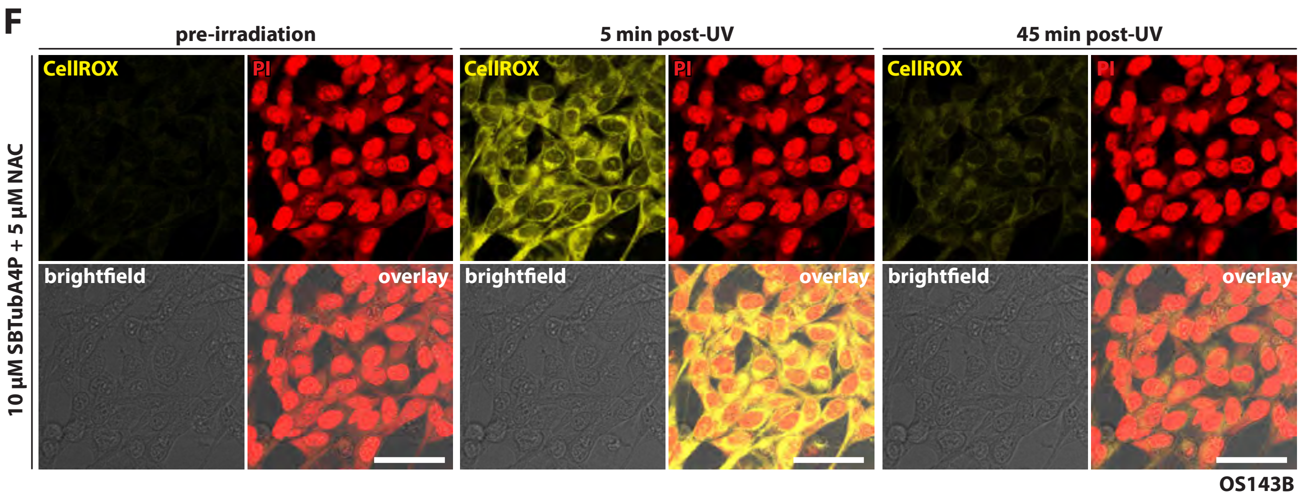
F
pre-irradiation
5 min post-UV
45 min post-UV
10 µM SBTubA4P + 5 µM NAC
CellROX
PI
brightfield
overlay
OS143B

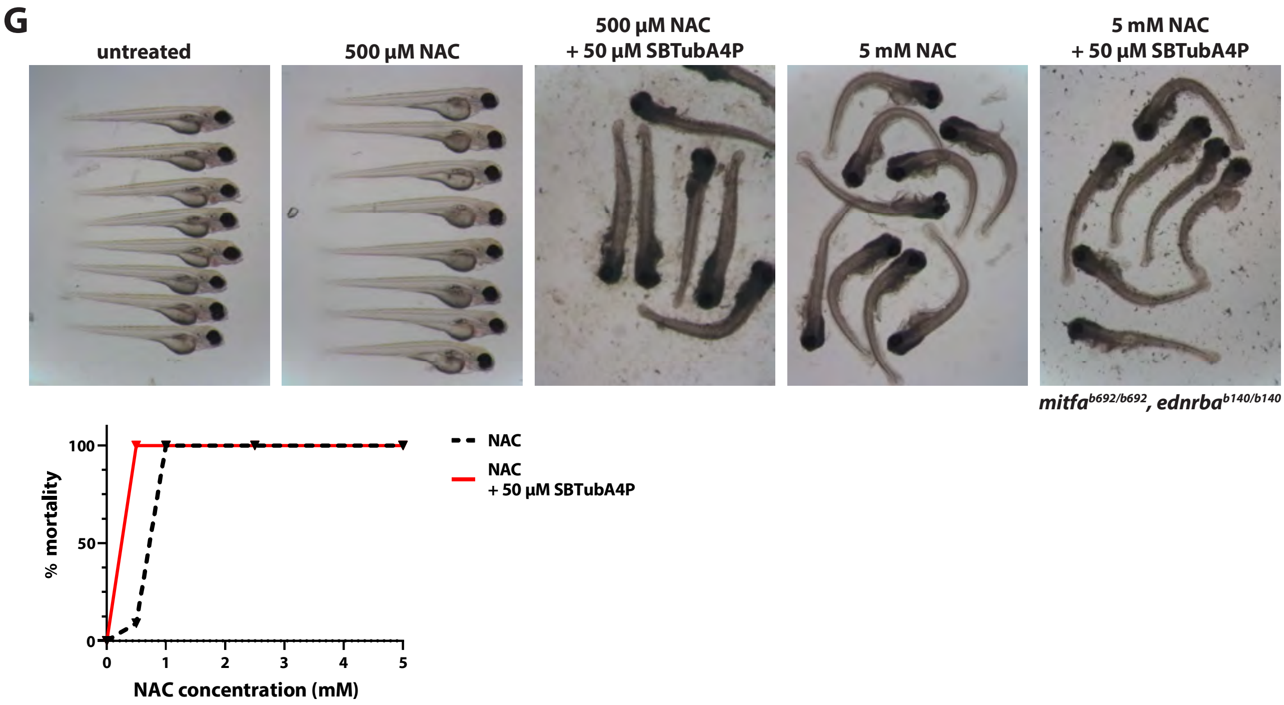
G
untreated
500 µM NAC
500 µM NAC + 50 µM SBTubA4P
5 mM NAC
5 mM NAC + 50 µM SBTubA4P
mitfa b692/b692, ednrba b140/b140
% mortality
NAC concentration (mM)
NAC
NAC + 50 µM SBTubA4P

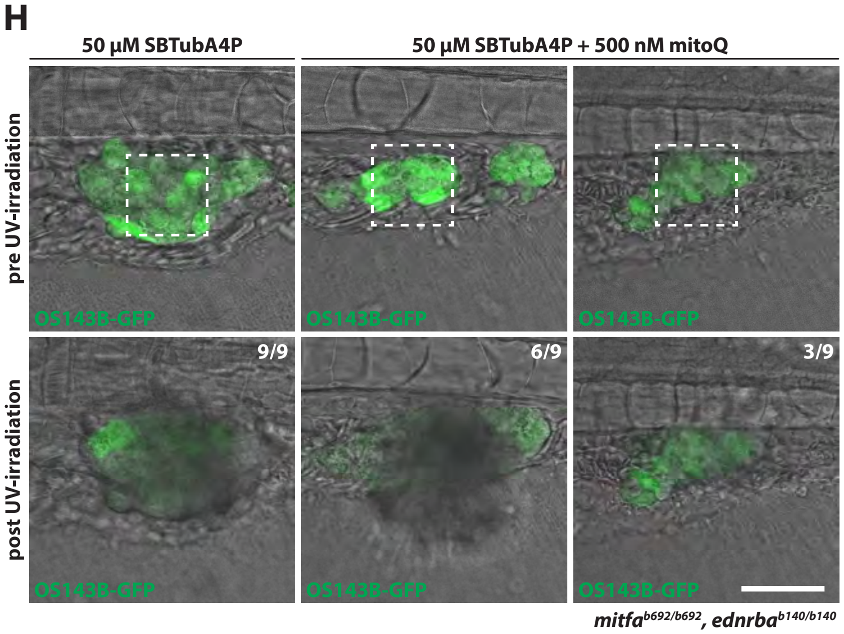
H
50 µM SBTubA4P
50 µM SBTubA4P + 500 nM mitoQ
pre UV-irradiation
post UV-irradiation
OS143B-GFP
9/9
6/9
3/9
mitfa b692/b692, ednrba b140/b140

Fig. S3. Mechanism of cell death induction

(A) CA4P and *Z*-SBTubA4P depolymerize microtubules but do not induce rapid cell death *in vitro*. OS143B-EB3-mNeon cells were treated with 10 µM *E*-SBTubA4P, 10 µM *Z*-SBTubA4P (pre-activated by UV irradiation before treatment), 100 nM CA4P or left untreated (control) for 1 hour before imaging. Representative confocal images from 2 independent experiments. Scale bar: 100 µm.

(B) UV-irradiation of *Z*-SBTubA4P but not CA4P leads to induction of cell death. OS143B-EB3-mNeon cells were treated with 10 µM *Z*-SBTubA4P or 100 nM CA4P for 1 hour before UV-irradiation for 5 minutes. Imaging was performed before and 15 minutes after UV treatment. Representative confocal images from 2 independent experiments. Scale bar: 50 µm.

(C) Line plots depicting the percentage increase of CellROX fluorescence area over the course of UV illumination in 3 separate experiments (CellROX area divided by GFP area, normalized to the respective baseline of CellROX area).

(D) SBTubA4P amplifies intracellular ROS levels in cancer cells *in vitro*. OS143B cells were treated with 10 µM SBTubA4P (lower panel) or left untreated (control – upper panel) for 1 hour before UV illumination in the presence of 5 µM CellROX Deep Red for 5 minutes. CellROX fluorescence (yellow) indicates the presence of ROS. Representative confocal images from 2 independent experiments. Scale bars: 25 µm.

(E) NAC mediates PI uptake without cell death. OS143B cells were treated with 5 µM NAC, PI or a combination of both 1 hour before imaging. The co-treated cells (right panel) take up PI in the absence of cell death. Representative confocal images. Scale bar: 100 µm.

(F) NAC protects cells from SBTubA4P-mediated killing despite PI uptake. OS143B were co-treated with 10 µM SBTubA4P with and without 5 µM NAC for 1 hour before UV illumination for 5 minutes in presence of 5 µM of CellROX Deep Red and PI. Representative confocal images for n = 2. CellROX fluorescence indicates ROS levels. Scale bars: 50 µm.

(G) NAC treatment is toxic to zebrafish larvae alone or combined with SBTubA4P. 4-dpf old zebrafish larvae were incubated with NAC (0, 500 µM, 1 mM, 2.5 mM or 5 mM) with or without 50 µM SBTubA4P overnight at 34° C under dark conditions. Toxicity (mortality) was assessed on the following day by counting dead and alive larvae. Representative images and mortality curve for n = 36-41 larvae (NAC only) and n = 5-8 larvae (NAC + SBTubA4P) per concentration.

(H) MitoQ treatment partially rescues SBTubA4P-mediated tissue damage. 2-dpf old zebrafish larvae were transplanted with OS143B-GFP cells as outlined in Fig. 3A. At 3 dpf, larvae were incubated with 50 µM SBTubA4P with or without 500 nM mitoQ for 1 hour before UV illumination. Dashed rectangles depict areas targeted for UV-irradiation (97 x 97 µm) by laser scanning for 5 minutes (every 10 seconds). Representative confocal images from 3 independent experiments (n = 9 per condition), showing both rescued (3/9) and non-rescued (6/9) larvae co-treated with mitoQ. Scale bar: 100 µm.

Disease Models & Mechanisms • Supplementary information

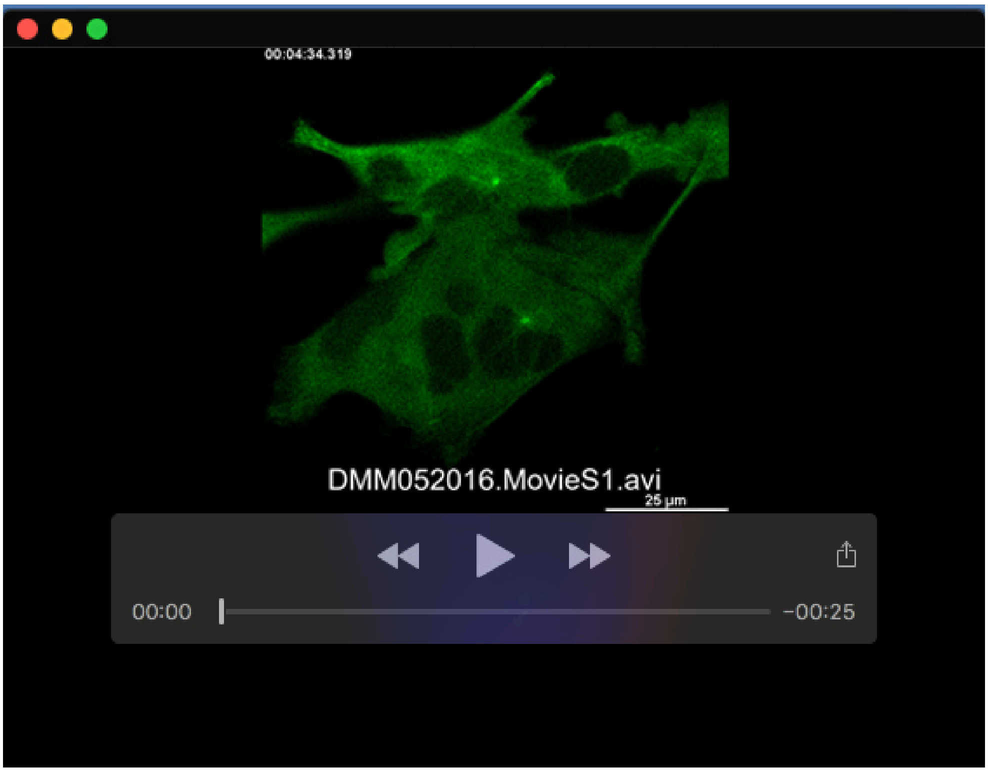

Movie 1. Depolymerization of microtubules after SBTubA4P UV-irradiation followed by recovery in OS143B cells (Fig. S1A). Images were taken every 5 seconds on a Leica SP8 WLL using a 40x objective.

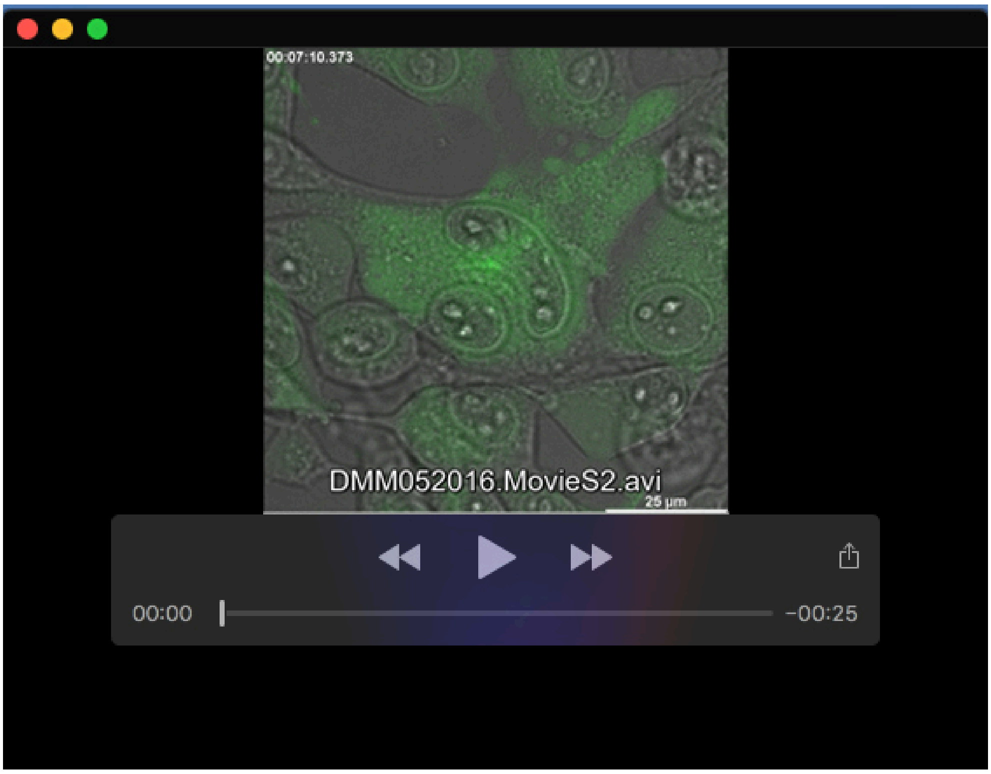

Movie 2. SBTubA4P-mediated killing of cells after repeated UV-irradiation for 5 minutes through rupturing of cell membranes as apparent by swelling and loss of eGFP fluorescence (see Fig. 1A). Images were taken every 14 seconds on a Leica SP8 WLL using a 40x objective.

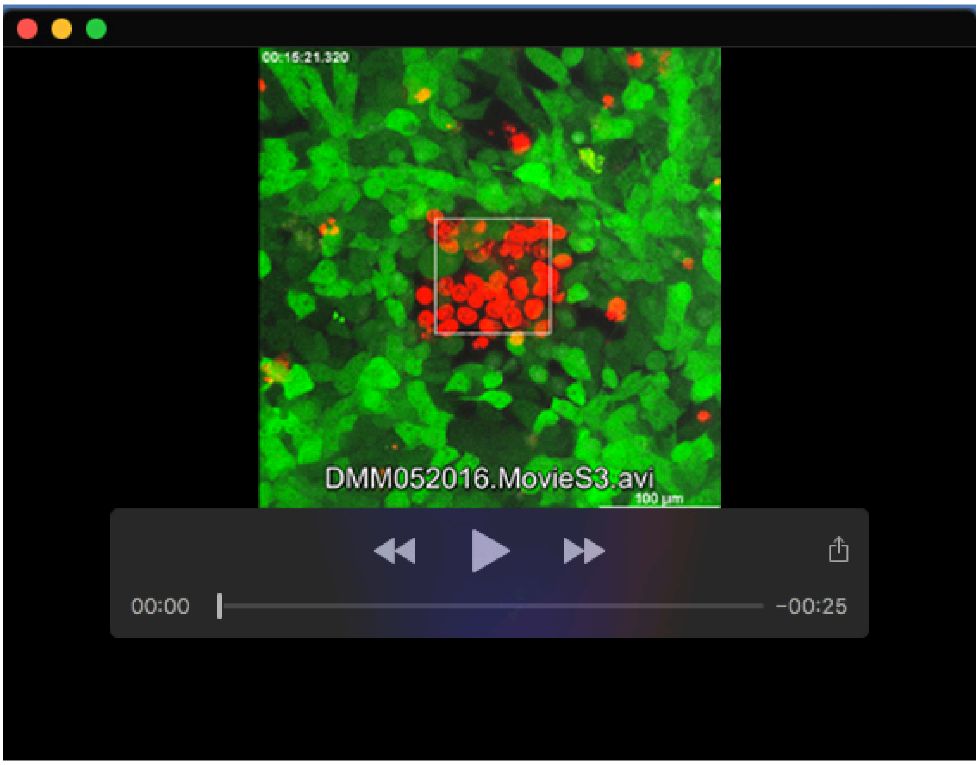

Movie 3. OS143B-eGFP cells dying within 30 minutes after SBTubA4P UV-irradiation in targeted ROI, visualized by PI live staining (see Fig. 1B). Images were taken every 5 minutes (maximum projection: 13 planes, 5 µm spacing) on a Leica SP8 WLL using a 40x objective.

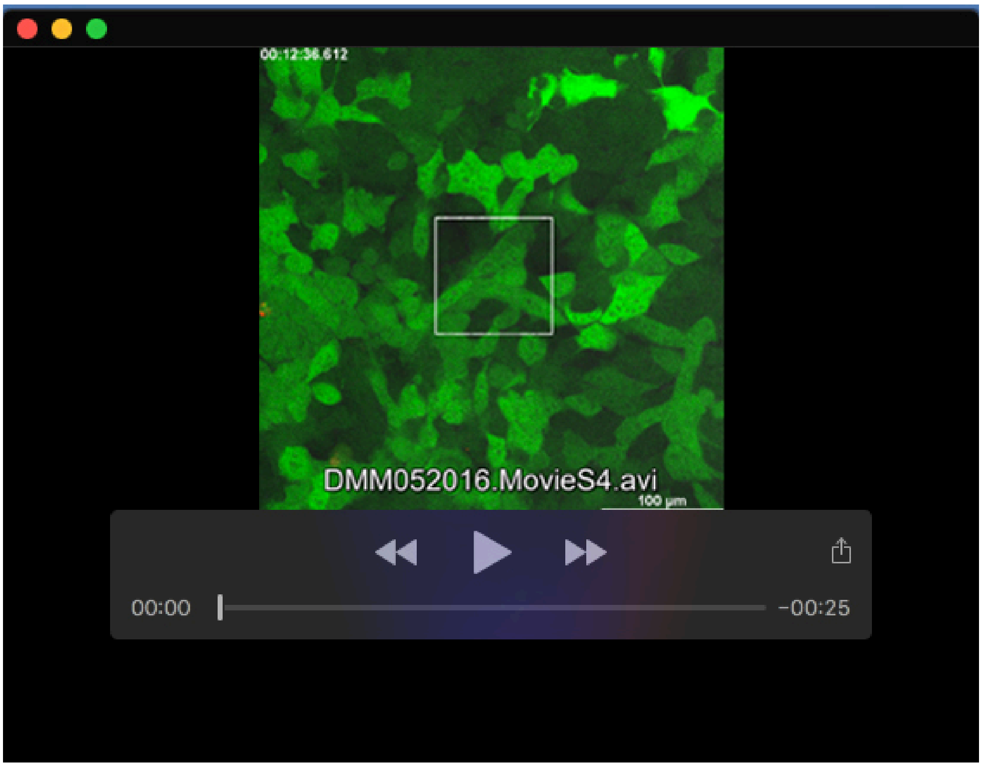

Movie 4. Time-lapse of untreated OS143B-eGFP cells after UV irradiation does not induce cell death (see Fig. 1B). Images were taken every 5 minutes (maximum projection: 13 planes, 5 µm spacing) on a Leica SP8 WLL using a 40x objective.

Disease Models & Mechanisms • Supplementary information

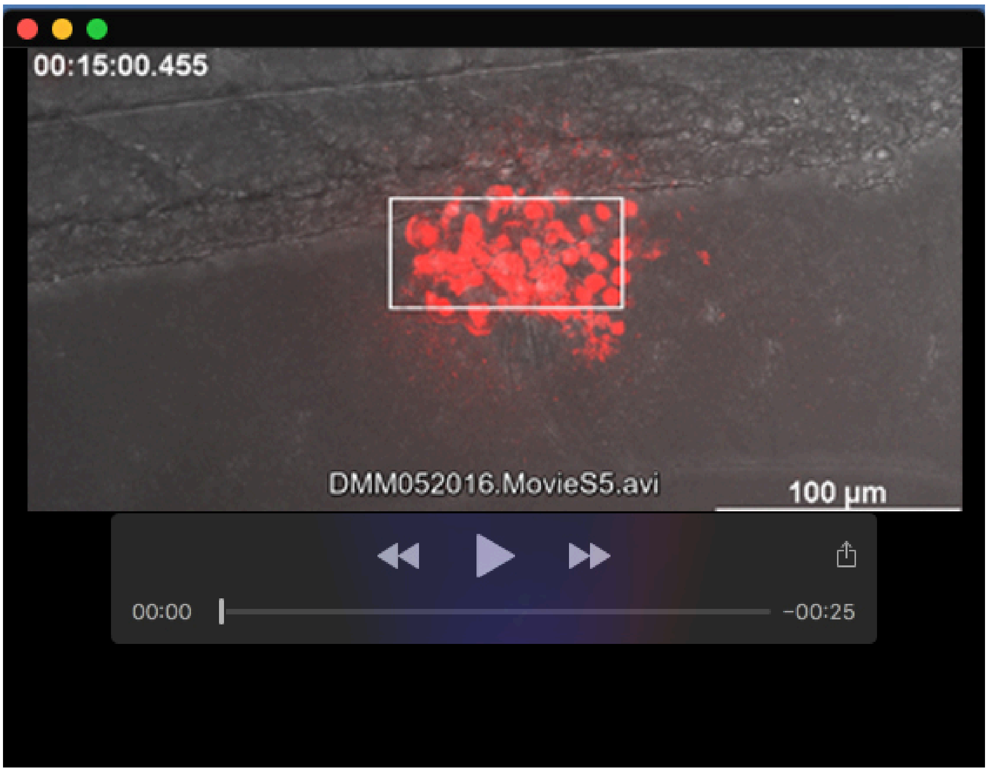

Movie 5. Induction of tissue damage in 50 µM SBTubA4P-treated zebrafish larvae at targeted ROI in the caudal fin fold as visualized by PI live staining (see Fig. 2B). Images were taken every 3 minutes (maximum projection: 22 planes, 5 µm spacing) on a Leica SP8 WLL using a 40x objective.

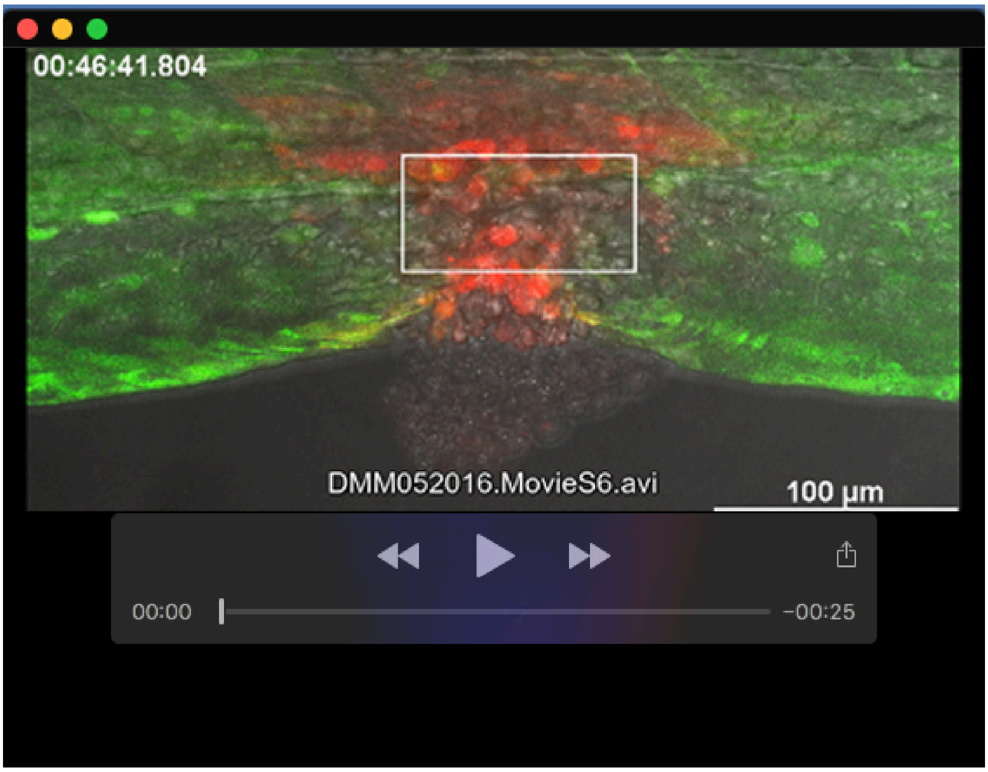

Movie 6. Demonstration of spatial precision of SBTubA4P-mediated tissue damage in zebrafish larvae ubiquitously expressing photoconvertible fluorescent protein Kaede. Extrusion of UV-irradiated tissues in the caudal fin fold of 50 µM SBTubA4P-treated larva (see Fig. 2C). Images were taken every 5 minutes 50 seconds (maximum projection: 22 planes, 5 µm spacing) on a Leica SP8 WLL using a 40x objective.

Disease Models & Mechanisms • Supplementary information

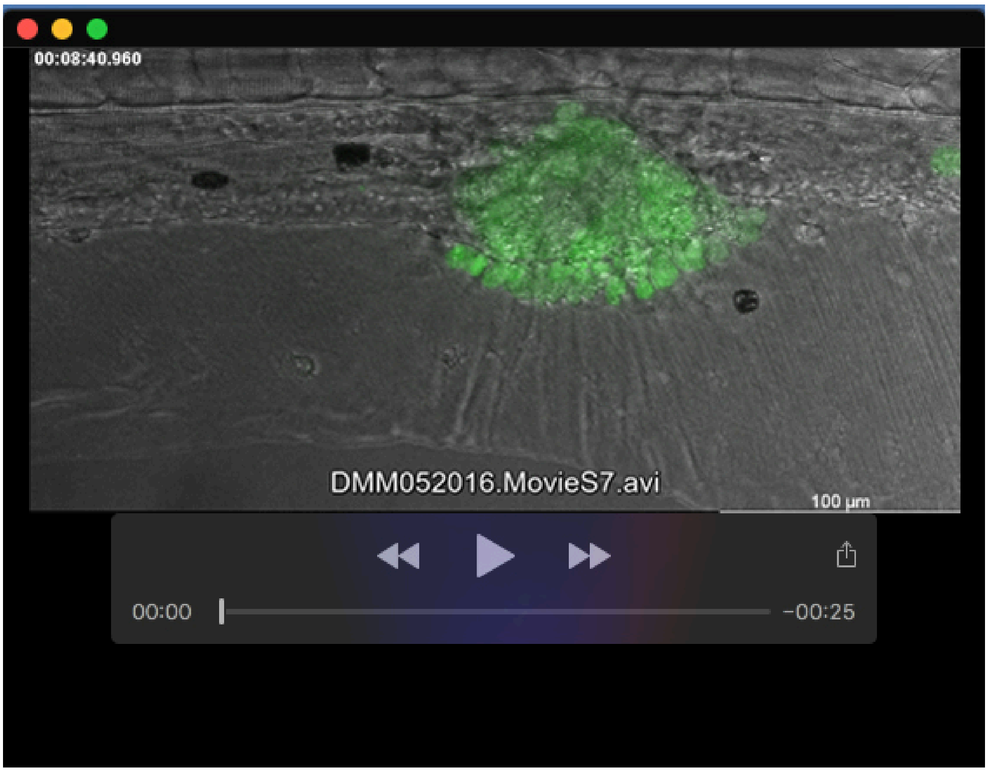

Movie 7. Induction of cell death and extrusion of UV-irradiated larval zebrafish tissue and xenografted OS143B-eGFP tumor cells in the tail region of 50 µM SBTubA4P treated larva (see Fig. 3B). Images were taken every 37 seconds (maximum projection: 14 planes, 3 µm spacing) on a Leica SP8 WLL using a 40x objective.

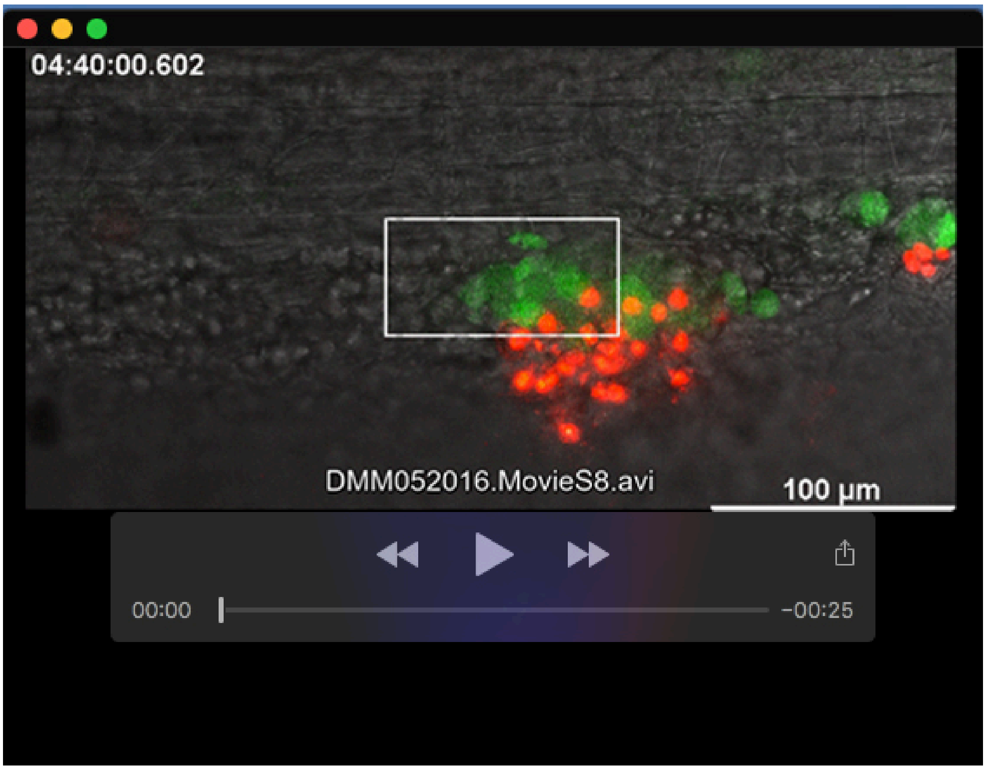

Movie 8. Precise eradication of tumor cells in the tail region of 50 µM SBTubA4P-treated zebrafish larvae visualized by PI uptake and loss of eGFP fluorescence in extruded cells (see Fig. S2C). Images were taken every 10 seconds (maximum projection: 9 planes, 5 µm spacing) on a Leica SP8 WLL using a 40x objective.

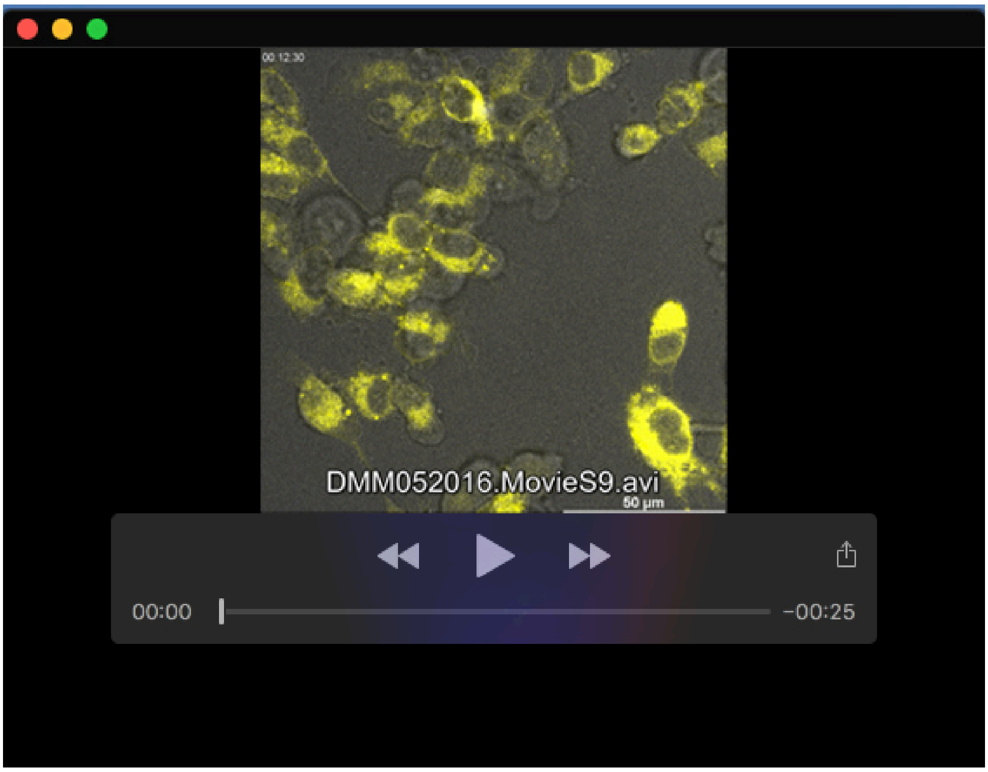

Movie 9. Induction of ROS generation followed by rapid cell death in UV-irradiated OS143B cells treated with 10 µM SBTubA4P, ROS visualized by CellROX (see Fig. S3D). Images were taken every 30 seconds on a Leica SP8 WLL using a 40x objective.

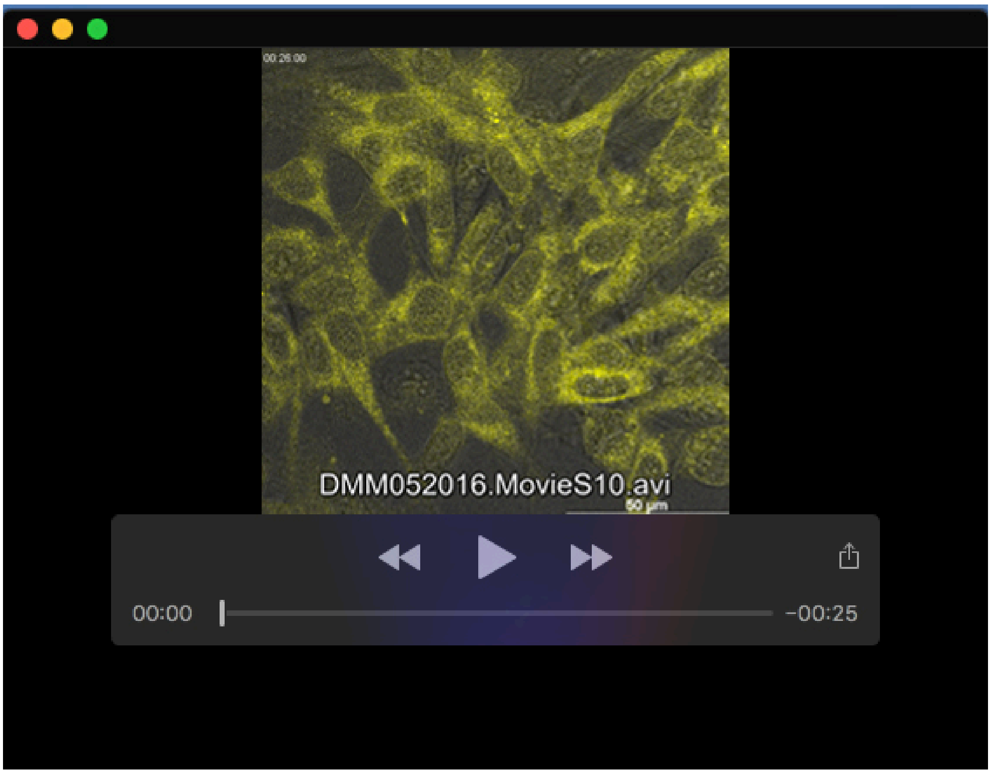

Movie 10. NAC (5 µM) protects 10 µM SBTubA4P treated OS143B cells from ROS-mediated killing (see Fig. 4C). Images were taken every 8 seconds on a Leica SP8 WLL using a 40x objective.
